# Self-Organizing Wireless Sensor Networks Solving the Coverage Problem: Game-Theoretic Learning Automata and Cellular Automata-Based Approaches †

**DOI:** 10.3390/s25051467

**Published:** 2025-02-27

**Authors:** Franciszek Seredynski, Miroslaw Szaban, Jaroslaw Skaruz, Piotr Switalski, Michal Seredynski

**Affiliations:** University of Siedlce, Institute of Computer Science, 08-110 Siedlce, Poland; franciszek.seredynski@uws.edu.pl (F.S.); jaroslaw.skaruz@uws.edu.pl (J.S.); piotr.switalski@uws.edu.pl (P.S.); michal.seredynski@uws.edu.pl (M.S.)

**Keywords:** collective behavior, adaptive cellular automata, learning automata, network coverage problem, self-organization, sensor networks, spatial prisoner’s dilemma

## Abstract

In this paper, we focus on developing self-organizing algorithms aimed at solving, in a distributed way, the coverage problem in Wireless Sensor Networks (WSNs). For this purpose, we apply a game-theoretical framework based on an application of a variant of the Spatial Prisoner’s Dilemma game. The framework is used to build a multi-agent system, where agent-players in the process of iterated games tend to achieve a Nash equilibrium, providing them the possible maximal values of payoffs. A reached equilibrium corresponds to a global solution for the coverage problem represented by the following two objectives: coverage and the corresponding number of sensors that need to be turned on. A multi-agent system using the game-theoretic framework assumes the creation of a graph model of WSNs and the further interpretation of nodes of the WSN graph as agents participating in iterated games. We use the following two types of reinforcement learning machines as agents: Learning Automata (LA) and Cellular Automata (CA). The main novelty of the paper is the development of a specialized reinforcement learning machine based on the application of (ϵ,h)-learning automata. As the second model of an agent, we use the adaptive CA that we recently proposed. While both agent models operate in discrete time, they differ in the way they store and use available information. LA-based agents store in their memories the current information obtained in the last *h*-time steps and only use this information to make a decision in the next time step. CA-based agents only retain information from the last time step. To make a decision in the next time step, they participate in local evolutionary competitions that determine their subsequent actions. We show that agent-players reaching the Nash equilibria corresponds to the system achieving a global optimization criterion related to the coverage problem, in a fully distributed way, without the agents’ knowledge of the global optimization criterion and without any central coordinator. We perform an extensive experimental study of both models and show that the proposed learning automata-based model significantly outperforms the cellular automata-based model.

## 1. Introduction

Wireless Sensor Networks (WSNs) are a fast-developing technology belonging to a broader group of information and communication technologies [1] currently applied in the Internet of Things. They are composed of a large number of tiny computer communication devices, called sensors, deployed in some areas. Their main duties are to sense a local environment and send the related information to a remote user who can make an appropriate decision, which after sending back will be realized by them. They have shown their potential in a number of applications, such as monitoring environmental parameters and threats (humidity, pollution, forest fire detection, etc.), healthcare monitoring, agriculture and industrial monitoring, military applications, and mission-critical systems. Many applications of Wireless Sensor Networks (WSNs), especially those termed as Ambient Intelligence (AmI), increasingly require not only sensing the surrounding environment but also providing intelligent responses based on them recognizing their own states. This approach limits the need for the costly communication with a remote user. Designing such systems is a complex task demanding solving a number of issues on different levels related to a single sensor node, a network of nodes, applications, etc. (see, e.g., [2]), and this is the subject of ongoing research.

This paper focuses on some issues related to designing fault-tolerant WSN-based mission-critical systems with some AmI abilities. We assume that monitoring is performed in a remote and difficult-to-access area and sensors are equipped with single-use batteries that cannot be recharged. From the Quality of Service (QoS) point of view of such a WSN, two closely related important issues exist as follows: how to perform effective monitoring (coverage) of an area and how to maximize an operational lifetime. These questions are usually solved by a remote user who delivers solutions to WSN, but we ask whether these problems can be solved by themselves, without  the need for asking a remote user for operational support each time.

After deploying the sensors, they should recognize their nearest neighbors with which they are meant to communicate and start making local decisions about turning their batteries on or off to monitor events. These decisions will directly influence the degree of area coverage, the amount of spending on sensors’ battery energy, and the network’s lifetime. One can notice that the lifetime maximization problem is closely related to the coverage problem. A group of sensors monitoring an area is usually redundant, i.e., more than one sensor covers the monitored targets, which creates some redundancy that can be exploited. Solving the coverage problem is crucial in solving the issue of the maximizing the lifetime of a WSN. In the paper, we will focus on the coverage problem.

This paper presents a novel approach to the problem of the coverage/lifetime optimization of WSNs based on self-organization. The notion of self-organization is usually considered a feature of complex systems that can achieve a specific global state called an emergent state (see, e.g., [3]) as a result of local interactions alone between single components of a system. Emergent behavior is very often used in the literature concerning Cellular Automata (CA) (see, e.g., [4]). In the area of LA (see, e.g., [5]), this feature is very often called collective behavior. In complex systems existing in real life, this notion is used more and more frequently to address specific issues. In our approach, via self-organization (called also collective behavior), we will assume that WSN sensors can be treated as rational agents; they know only some information about their sensors-neighbors and they use it for making some decisions. In the result of such an interaction, they can achieve certain states corresponding to a global solution, i.e., solving the coverage problem without any central coordinator.

The main novelty of this paper is working out of the self-organizing system which uses the concept of (ϵ,h)-learning automata (LA) [6,7], and it is an extended version of the recently published paper by Seredynski et al. (2024) [8]. The paper continues and extends the line of research proposed in [9], where a self-organizing approach to solve the coverage problem with the use of adaptive CA (also called a second order CA) was used. Both papers use a recently proposed game-theoretic framework [10], assuming interactions between players participating in the Spatial Prisoner’s Dilemma (SPD) game, which serves as a multi-agent platform used to solve the coverage problem in WSNs. We experimentally prove in this paper that the new approach presented, based on the application of LA-based agents, significantly improves the quality of found solutions, and this approach significantly outperforms the approach based on the application of CA-based agents.

Our approach based on self-organization contrasts with other approaches currently used to solve the coverage/lifetime maximization problem. Because these problems are known as NP-complete [11], centralized algorithms are oriented either on the delivery of exact solutions for specific cases (see, e.g., [12]) or applying heuristics or metaheuristics to find approximate solutions (see, e.g., [13,14]). The main drawback of centralized algorithms is that they assume the availability of complete information about the problem and the availability of computing power to find a schedule of sensors’ activities, which can be practically carried out in a remote user site, outside the WSN, and the solution must be delivered to it before beginning the operation. Therefore, distributed algorithms with different forms of partial information about the problem have become increasingly popular because they simplify control over WSNs and assume some of the reactivity of sensors in real-time (see, e.g., [15,16]). The full independence of the computational power of a remote user, scalability, and the entire operation of a WSN in real time can establish self-optimizing systems, which can rely on the computational power of the sensors alone. The need for such systems has been recognized in recent years across many industrial systems and is the subject of various current studies (see, e.g., [17,18]).

To the best of our knowledge, our paper [9] is the first paper to propose using principles of self-organization in WSNs. We presented a classification and review of the current approaches in the literature that recognize centralized algorithms, distributed algorithms, and self-organizing algorithms in solving the coverage/lifetime problems in WSNs, and below, we give some new literature notes concerning these issues. As we have already noted the term self-organization did not exist in the current literature concerning WSN, it was nevertheless used in other domains such as mobile communication [19], load balancing in organic computational systems [20], or distribution of services in grid and ubiquitous systems [21].

A recent paper [22] was devoted to self-organizing WSNs. In this paper, network self-organization is formulated as a requirement for achieving the automatic configuration and management of the entire network through autonomous collaboration and coordination among nodes, thereby providing conditions for routing. Network self-organization is based on the following two processes: topology discovery and network clustering. In the topology discovery stage, a small number of anchor nodes are identified to meet discovery needs while minimizing discovery complexity and reducing network costs. In the clustering stage, nodes locally communicate and elect clusters in a way that balances network energy.

This paper [23] presents some new concepts for designing distributed algorithms for WSNs. It presents a hybrid decentralized algorithm called DACYCLEM, whose purpose is to maximize the coverage and lifetime in mobile WSNs by organizing the work of sensors to monitor the environment as long as possible and cover a large surface area. This approach is based on building a connected dominating set and using attractive and repulsive forces for sensor movement to maintain network connectivity.

Most of the recent papers concerning coverage/lifetime optimization in WSNs are centralized algorithms, where authors apply different usually bioinspired metaheurisics [24] to find an approximate solution for a considered problem. An energy-efficient coverage area optimization technique for WSNs using a hybrid algorithm, called the MOFAC-GA-PSO (Minimum Overlapped Full Area Coverage using hybridized Genetic Algorithm-Particle Swarm Optimization) algorithm, is proposed in [25]. In [26], the authors proposed an enhanced version of the Gray Wolf Optimizer algorithm for enhancing resource utilization, such as reducing the number of nodes, by maximizing the coverage rate and maintaining connectivity.

Hybrid Lion Swarm Optimization is proposed in [27] to solve the coverage problem in 3D space. Ref. [28] presents an optimized coverage strategy for WSN nodes based on path loss and false alarm probability. In [29], the coverage of a circular area shape WSN using a disk-shaped deployment strategy is proposed. An algorithm based on concentric hexagonal tessellations and the concept of coverage contribution area for randomly deployed nodes in the field of interest has been proposed to generate the maximum number of disjoint-independent subsets of sensor nodes as an optimized solution to the coverage problem, along with maximizing the WSN lifetime (see [30]). In [31], the authors proposed an optimization method using an artificial bee colony algorithm with a teaching strategy based on teaching-learning-based optimization to obtain WSN coverage.

A multi-strategy improved sparrow search algorithm for coverage optimization in WSNs was proposed in [32]. The paper [33] proposes a coverage optimization method based on an improved hybrid strategy weed algorithm. Many other algorithms were recently proposed and used for solving the coverage problem as follows: the marine predator algorithm (IMPA) in [34], modified marine predator algorithm (MMPA) in [35], improved COOT bird algorithm (COOTCLCO) in [36], improved Archimedes optimization algorithm (EAOA) [37], and improved honey badger algorithm (IHBA) [38]. The coverage optimization technique called the Voronoi-Glowworm Swarm Optimization-k-means algorithm (see [39]) was used to enhance the coverage area with a minimum number of active nodes.

More and more authors apply a multi-objective optimization in WSNs; more specifically, a multiobjective optimization algorithm for WSNs, based on Improved Particle Swarm Optimization-Increment of the Ratio of Coverage Rate to Move Distance, aimed at maximizing of network node coverage rate, was presented in [40]. Recent papers [41,42,43] have present reviews concerning the multiobjective algorithms applied for WSNs, and [44] presents a review concerning applications of machine learning algorithms in WSNs.

The main highlights of the paper are the following:

- A game-theoretical framework based on a variant of a Spatial Prisoner’s Dilemma game is presented;
- A multi-agent system interpretation of WSNs is given;
- A payoff function reflecting the global goals of the coverage problem is developed;
- An analysis of the Nash equlibria of the game and their linking with a global optimization criterion related to the coverage problem is presented;
- Two models of agents are presented, the LA-based model and the CA-based model;
- Two variants of self-organizing algorithms solving the coverage problem are presented;
- It is experimentally proven that the LA-based variants of self-organizing algorithms significantly outperform the CA-based variants.

The structure of the paper is as follows. The next section presents the problem of a coverage optimization in WSNs. Section 3 presents an idea of the conversion of an instance of the coverage problem into a WSN graph. In Section 4, a multi-agent system for a self-organizing algorithm solving WSN coverage optimization is presented. The payoff function of a SPD-like game used for WSN optimization is shown in Section 5. The concept of (ϵ,h)-LA used as players in the game is proposed in Section 6. Section 7 presents the LA-based version and the CA-based version of self-organizing algorithms solving the coverage problem online. Section 8 discusses the relationship between concepts of a global solution and Nash equilibria. Section 9 presents the results of the experimental study, and the last section contains the conclusions.

## 2. Coverage Problem in Wireless Sensor Networks

**Figure 1:**
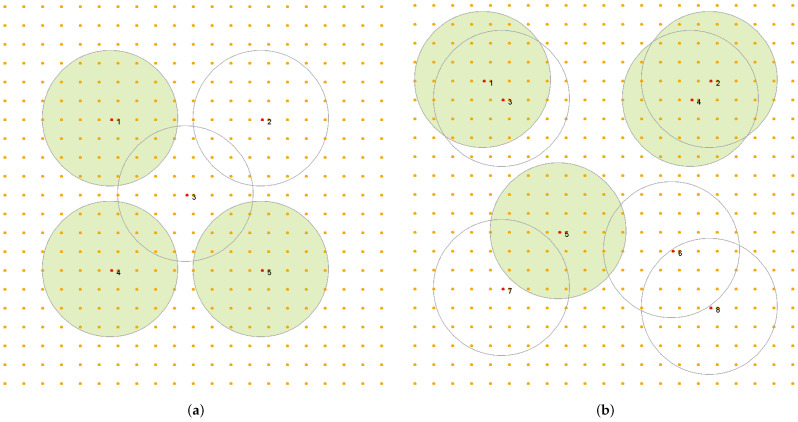
An example of a monitored area containing 441 PoI: (**a**) a WSN 5 consisting of 5 sensors with $R_{s}=18$ m, (**b**) a WSN 8 consisting of 8 sensors with $R_{s}=18$ m.

We assume that an area of size $L_{1}$ × $L_{2}$ $m^{2}$ should be monitored by a sensor network $S=\{s_{1},s_{2},\ldots ,s_{i},\ldots ,s_{N}\}$ consisting of *N* sensors deployed over this area. More specifically, the area is represented by *M* “Points of Interest” (PoI), which regularly cover the area and should be monitored. Each sensor has a non–rechargeable battery of capacity batt_capacity and can monitor PoIs in a sensing range $R_{s}$ if its battery is turned on. Figure 1 shows an example of such an area with $L_{1}$ = $L_{2}$ = 100 m and M=441 PoI (in orange), where a WSN 5 consisting of N=5 sensors with $R_{s}=18$ m (see Figure 1a) or a WSN 8 consisting of N=8 sensors also with $R_{s}=18$ m was deployed (see Figure 1b). Some sensors of both WSNs are currently turned on and monitor the corresponding areas (in green).

It assumed that a QoS measure exists evaluating the quality of a WSN performing monitoring. As such, we accept the coverage value defined as the ratio of the number of PoIs covered by active sensors to the whole number *M* of PoIs. The coverage *q* of a target area can be denoted as

(1) $$q_{j}=\frac{M_{obs_{j}}}{M}.$$

A desirable objective is to preserve the complete area coverage, but sometimes, it may be more practical to achieve a predefined coverage rate that is just high enough. Therefore, we assume that this ratio should not be lower than some predefined requested value $q_{r}$$\left(0<q_{r}\leq 1\right)$.

Figure 1 shows two coverage examples of the monitored area related to using different WSNs. When WSN 5 is used and three sensors are turned on (n_on(s)=3), as shown in Figure 1a, we have to do with a solution $s_{19}=\left(s_{1}=1,\;s_{2}=0,\;s_{3}=0,\;s_{4}=1,\;s_{5}=1\right)$, with a corresponding value of coverage *q* being equal to 0.25. The solution $s_{216}=\left(11011000\right)$, presented in Figure 1b, uses more turned on sensors (n_on(s)=4), but the corresponding coverage q=0.27 is only slightly greater than the solution shown in Figure 1a. This is because WSN 8 contains some potentially redundant sensors, i.e., sensors deployed at similar locations in the monitored area, like sensors 1 and 3 or sensors 2 and 4. On the one hand, such situations offer the possibility of decreasing the number of sensors that need to be turned on, which minimizes the energy costs of coverage. However, on the other hand, it makes searching for an effective solution more difficult.

The coverage problem can be stated as follows. Find a solution $s=(s_{1},\;s_{2},\;\ldots ,\;s_{i},\;\ldots ,\;s_{N})$ with corresponding coverage values q(s) and the number n_on(s) of sensors turned on such that it fulfills the following requirement: (a) the number n_on of sensors turned on is minimal, (b) a value of the coverage fulfills $q\geq q_{r}$, and (c) this *q* value is maximal.

We have to deal with a combinatorial optimization problem, which can be described by a proposed function that should be maximized as follows:

(2) f(q(s),n_on(s),qr)=q(s)+N−n_on(s),ifq(s)≥qrq(s),ifq(s)<qr.

It can be shown that this function univocally assigns values to solutions in such a way that a maximal value of it corresponds to a solution (or solutions) that provides a maximal value of *q* meeting the requirement $q\geq q_{r}$, under the minimal value of n_on sensors turned on (see Section 8 for further discussion). The space of possible solutions to the coverage problem exponentially increases with the growth of *N*. For N=5, the solution space contains 32 solutions; for N=8, the number of solutions equals 256 and grows very fast as *N* increases.

As mentioned, the problem is NP-hard; therefore, for realistic sizes of *N*, we can rely only on metaheuristics. These (see, e.g., [24]) belong to a category of centralized methods. Applying them assumes the availability of complete knowledge about an instance of the problem, such as the sensor locations, levels of their batteries, etc. This means that the problem can be solved only online, at the site of a remote owner responsible for monitoring an area and having access to the necessary computational power.

However, we are interested in something other than a centralized approach. We want to solve the coverage problem online, in real time, using only a tiny amount of computational power and the communication possibilities of WSN sensors. A potential solution algorithm should work in a monitored area in real time, fly in real time, react quickly to potential changes in the values of sensor parameters, and have small requirements for the available computational and communication facilities of a WSN. Therefore, in this paper, we focus on working out a variant of a distributed algorithm to solve the coverage problem by self-optimization.

The first step in this direction is converting a WSN instance of a coverage problem into a WSN interaction graph. Such a WSN interaction graph will be used as the core of a multi-agent system aimed at solving a coverage problem online.

## 3. Convertion of WSN Instance into WSN Interaction Graph

To apply a multi-agent approach to solve a coverage problem in online mode, we need to represent a given instance of the problem related to monitoring an area by a WSN consisting of *N* sensors, deployed in some locations of the monitored area by a WSN interaction graph. The conversion is based on the principle in [9] that supports two nodes of a WSN graph are connected if they have at least one common PoI within their sensing range $R_{s}$ in a corresponding WSN.

**Figure 2:**
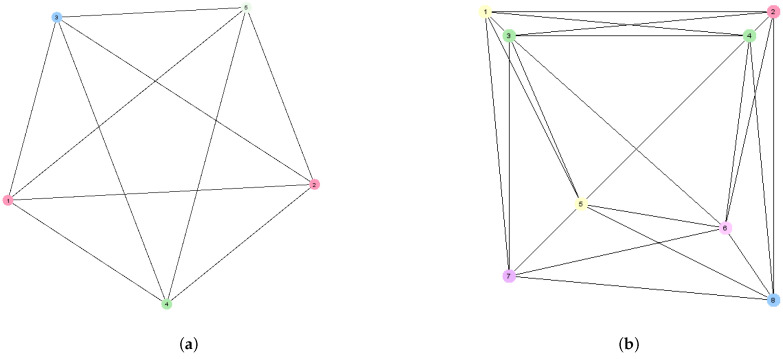
Convertion of a WSN instance of a coverage problem into a WSN interaction graph: (**a**) a WSN graph corresponding to WSN 5 with $R_{s}=35$ m, (**b**) a WSN graph corresponding to WSN 8 with $R_{s}=35$ m.

Figure 2 shows the WSN interaction graphs for the WSN instances presented in Figure 1 under the assumption that sensing range $R_{s}=35$. Figure 2a shows that the WSN interaction graph representing the instance from Figure 1a that contains five nodes, each corresponding to a sensor from the corresponding instance. The degree to which each node corresponds to the number of neighbors of each sensor generally depends on the value of $R_{s}$, and in this case, each sensor has four neighbors. Figure 2b shows that the WSN interaction graph representing the instance from Figure 1b contains eight nodes. The graph is irregular, with nodes ranging from 5 to 7.

## 4. Multi–Agent System for Online WSN Coverage Optimization

We assume that each node of a WSN interaction graph is controlled by an agent $A_{i}$ of a multi-agent system consisting of *N* agents. Each agent has two the following alternative decisions (actions): $\alpha_{i}=0$ (battery is turned off) and $\alpha_{i}=1$ (battery is turned on), where a decision unit responsible for making a corresponding decision is assigned to it. There exist different agent coordinations, with their decisions oriented towards reaching a common goal. In our approach, we assume that the interaction of the agents is based on a game-theoretic model, which is a variant of the Spatial Prisoner’s Dilemma (SPD) game [10], related to the WSN coverage optimization problem [9]. In this game-theoretic model, we assume that all agents make discrete-time decisions regarding the activation of their batteries using specific rules/strategies.

We assume that the following set of socially interpreted rules is available to the agent-players:

- *all C*: always cooperate (C), corresponding to turning on the battery ($\alpha_{i}=1$);
- *all D*: always defect (D), corresponding to turning off the battery ($\alpha_{i}=0$);
- *kD*: cooperate until no more than *k* neighbors defect, otherwise defect;
- *kC*: cooperate until no more than *k* neighbors cooperate, otherwise defect;
- *kDC*: defect until no more than *k* neighbors defect, otherwise cooperate.

One can observe that the first two rules do not consider player-neighbor decisions. The remaining three rules of a given agent-player take into account the decisions of player-neighbors from a previous game. Selecting a rule to turn on/off a battery depends on the algorithm of the player decision unit. While in our previous study [9] we were using a second order CA, in this paper, we propose using a new original reinforcement learning algorithm called (ϵ,h)-LA.

## 5. Payoff Function of a SPD-like Game for Coverage Optimization Problem

It is assumed that each agent-player knows the value of a global parameter, a requested coverage $q_{r}$, and they considers this value a local value $q_{r}^{i}=q_{r}$ which must fulfilled. Each player participates in an iterated SPD-like game [9] consisting of an unknown for them number of games. Each game is conducted in discrete moments of time t=1,2,…,T, where *T* is known only for an organizer (a remote user of WSN) of the iterated game. An agent will decide on C or D and obtain a payoff that depends on their actions and those of their neighbors alone, defined by a WSN graph. The neighbors of a given player can be considered a virtual player-opponent. A value of a local coverage $q_{curr}^{i}$ is the result of a game of the *i*-th player with their virtual opponent. Their payoff in a game depends on whether their current $q_{curr}^{i}$ is below or above the requested $q_{r}^{i}$. The payoff function of a player is given in Table 1.

The payoff function assigns values to the *i*-th player in the following way:

(a) If they “turn off battery” then they calculate their local value of coverage $q_{curr}^{i}$ using information about the common PoI with their neighbors. If this value $q_{curr}^{i}\geq q_{r}^{i}$ then they receive a payoff equal to *b*. Otherwise, a payoff equal to *a* is obtained;
(b) If they “turn on battery” then they calculate what would be their value of $q_{curr}^{i}$ (denoted as $q_{curr}^{i-off}$) if in fact they would have “turned off” their battery. If $q_{curr}^{i-off}<q_{r}^{i}$, then they receive a payoff equal to *d*. Otherwise, a payoff equal to *c* is obtained.

The proposed payoff function transforms the global optimization criterion (see Equation (2)) stated in Section 2 for the coverage problem into local optimization goals of players. The payoff function balances payoffs for battery energy spending expressed in the number of sensors n_on and payoffs for fulfillment of the requested coverage $q_{r}$ for different permutations of sensors turned on under a given n_on.

We assume that players are rational and act in such a way as to maximize their payoff defined by the payoff function. However, we are not interested in players’ payoffs but in the evaluation of the level of collective behavior of the system. As a measure of the collective behavior of the system, we will use an external criterion (not known for players), the average total payoff (ATP) $\bar{u}\left(\right)$, expressed as follows:

(3) $$\bar{u}\left(s_{1},s_{2},\ldots ,s_{i},\ldots ,s_{N}\right)=\frac{1}{N}\sum_{i=1}^{N}u_{i}\left(A_{i}\left(s_{i}\right),A_{i}^{virtual}\left(s_{i_{1}},s_{i_{2}},\ldots ,s_{i_{r}}\right)\right),$$

where $u_{i}\left(\right)$ is the payoff of an agent-player $A_{i}$ in a game with a virtual player $A_{i}^{virtual}$, and $i_{r}$ is the number of neighbors of a player $A_{i}$ corresponding to their opponent (player $A_{i}^{virtual}$).

Game theory predicts that players’ behavior in non-cooperating games is oriented towards achieving a Nash equilibrium (NE). At this point, we call the price of a NE the value of the ATP. The game can have many NE points with different ATPs. We call the NE with the highest ATP the *maximal price point* (MPP). We ask whether we can expect from the players of such a behavior, that while they attempt to reach a NE, at the same time, the ATP of the whole set of players is maximized, i.e., MPP is reached. Such a behavior depends on many factors, and one of them is an applied model of a player making decisions in a game. In this paper, we examine the collective behavior of players modeled by (ϵ,h)-LA.

## 6. (ϵ,h)-Learning Automaton and Deterministic Environment

Learning automata are reinforcement learning algorithms first proposed by [5] and further extended and studied by [6,45,46] and many others [47,48,49]. Their distinctive feature is their ability to work in random environments and adapt to such conditions. An idea of LA working in a deterministic environment called ϵ-automaton was presented in [46] (see also [6]), with a comment saying that such an idea seems to make sense only if a deterministic environment can be randomized. However, it was shown in [7] that the concept of ϵ-automaton is useful in game-theoretic models related to variants of PD game with deterministic environments. In this paper, we extend this idea and propose a version of (ϵ,h)-LA suitable for an SPD-like game models, aimed at solving coverage problems in WSN.

**Figure 3:**
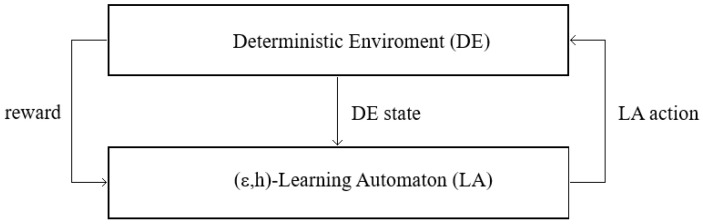
Concept of the (ϵ,h)-learning automaton.

Figure 3 presents the proposed general concept of (ϵ,h)-LA working in a deterministic environment (DE). LA represents a player participating in an SPD-like game, and the DE can evaluate LA’s actions. It is assumed that the number of LA-players is greater than 1, but a given LA does not directly interact with other LA but interacts with them via the DE. It is assumed that the LA player takes an action and sends it to the DE, which calculates a value of the payoff (reward) related to the SPD game and returns it to the LA. The DE may send LA some additional information (DE state) concerning, e.g., the actions taken by their neighbors. LA update their state, considering the received reward and information about the DE state, and produce a new action. The goal of the automaton is to maximize the rewards obtained from the DE.

**Figure 4:**
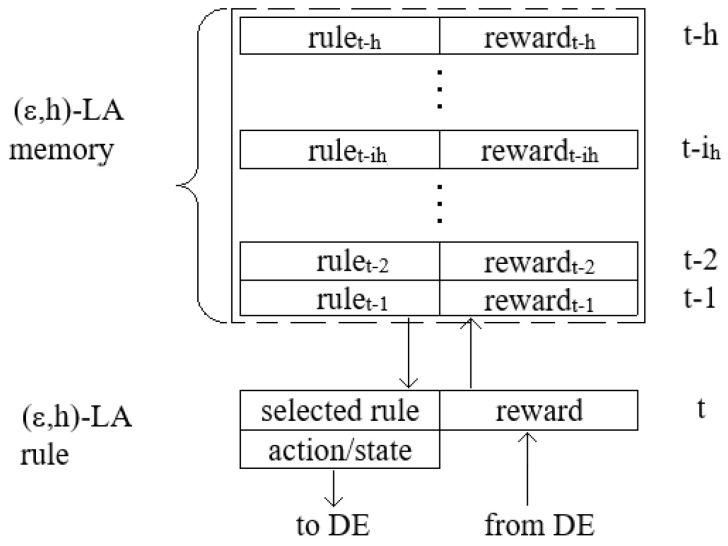
Proposed (ϵ,h)-learning automaton.

Figure 4 presents the proposed (ϵ,h)-LA construction details. Each LA possesses a set of the five available rules presented in Section 4 as follows: *all C*, *all D*, *kD*, *kC*, and *kDC*. In an operating mode of the system, it can use a subset of these rules of size *m* (0<m≤5). It has a memory of length *h*, where it stores pairs ($rule_{t}-i_{h},reward_{t}-i_{h}$) from the last *h* moments of discrete time $t-1,t-2,\ldots ,t-i_{h},\ldots ,t-h$ of its operation. Each pair contains information about a rule selected by LA at discrete time $t-i_{h}$ and the corresponding rewards for executing this rule.

An (ϵ,h)-LA is a reinforcement learning machine that can be applied to solve various problems requiring adaptation. In general, the operation of an (ϵ,h)-LA in a single discrete unit of time *t* can be described as shown in Algorithm 1.
**Algorithm 1** (ϵ,h)-LA operation in a single discrete unit of time *t*  1:*x* ← rand (0,1)  2:if x≤ϵ then $rule_{t}$ ← select with probability 1/m a rule from available set of rules  3:else  4:$rule_{t}$ ← select from rules stored in memory the rule with the highest value of reward  5:apply selected rule $rule_{t}$  //application dependent step  6:calculate $reward_{t}$ associated with the selected $rule_{t}$  //application dependent step  7:remove from memory the oldest pair (rule,reward) and store the new pair $\left(rule_{t},reward_{t}\right)$

First, a random value of *x* is generated (line 1). Depending on this value one of two options for selecting a new rule is realized as follows: with a probability ϵ, a rule from the available set of rules is selected (line 2), and with a probability 1−ϵ, a rule with the highest reward is selected from rules stored in the memory (line 4). Next (line 5), the selected rule is executed. The realization of this step depends on the problem to be solved, and in our case, the state of a corresponding sensor battery will be changed by performing the action turn ON or turn OFF (see Algorithm 1). The executed rule needs to be evaluated by assigning to it some reward (line 6), but this step is also application-dependent (see Algorithm 2). A record with the selected rule and a corresponding reward must be stored in the LA memory (line 7), but before that, the oldest record with pair (rule,reward) is removed.
**Algorithm 2** Coverage optimization by self-organization with use of (ϵ,h)-LA  1:Convert WSN instance into WSN graph  2:Assign LA-based agent $A_{i}$ to corresponding sensors $s_{i}$—nodes of WSN graph  3:Select a subset of rules allowed to use by agents and set allowed range of changes of parameter *k*  4:With a user-predefined probability turn on the battery states of the sensors, and send info about the battery state to neighbors  5:Perform a pregame consisting of *h* games to fill in memories of automata  6:**while** termination condition NOT TRUE **do**  7:    each LA-based agent selects their new rule: with a probability 1 − ϵ selects from their memory the rule with the highest reward and with a probability ϵ selects randomly a rule from available for agents set of rules  8:    each agent-player uses their current rule to set a new battery state  9:   each agent sends/receives to/from their neighbors information about a new state of battery and calculates a payoff according to Table 110:   each agent updates their memory: removes from the memory the oldest record and stores a new record with the pair (rule,reward)11:   global performance characteristics of the multi-agent system—coverage *q*, and the number of sensors turned on n_on are observed12:**end while**

**Figure 5:**
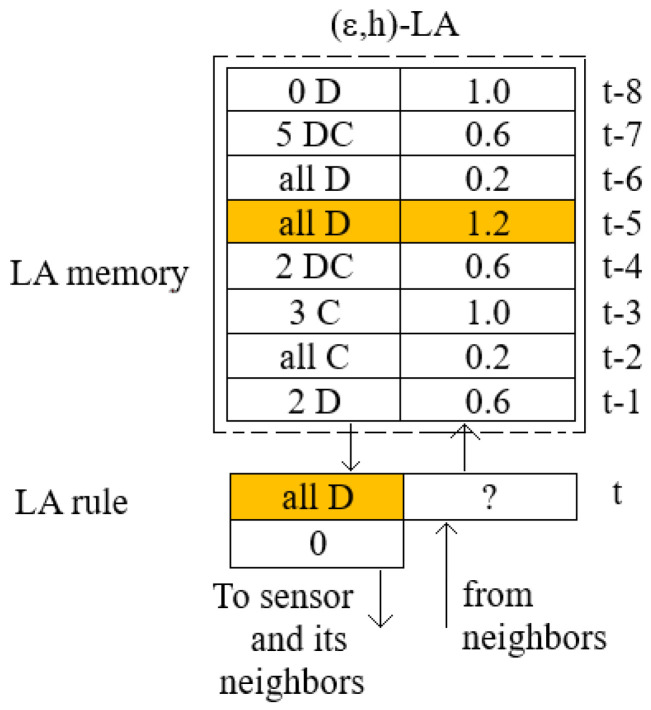
Example of (ϵ,h)-learning automaton with h=8.

Figure 5 shows an example of (ϵ,h)-LA with a memory size h=8 corresponding to an agent-player $A_{i}$ participating in solving the coverage problem. The LA memory contains actions (rules/strategies) applied by LA in a recent window time of a length 8 with corresponding rewards. Let us assume that at the current time *t* the LA searches for a new decision-rule and it is carried out according to line 4 of the Algorithm 1. One can easily observe that the winning action is the strategy *all D* because a reward associated with it equal to 1.2 is the highest. The strategy performs the action $\alpha_{i}=0$ (battery is turned off). Information about the winner strategy and the result of its execution are sent to the agent-neighbors, and the agent $A_{i}$ also receives similar information from their neighbors (see Algorithm 2). Due to this information, a reward corresponding to the applied rule *all D* can be calculated and stored in the memory.

The system requires a pregame phase to fill in the content of the memory. In order to achieve this, agents participate in an iterated pregame consisting of *h* rounds (iterations). In each round, each LA-based agent selects, with the probability 1/m, a rule from the set of rules available in the game and uses it in the game. The selected rule, together with a received reward, is stored in the memory.

## 7. Self-Organizing System Solving the Coverage Problem

### 7.1. Learning Automata-Based Approach

Our approach to solving the coverage optimization problem by self-organization using the SPD-like game and (ϵ,h)-LA can be summarized by Algorithm 2. It consists of three parts as follows: preparatory steps to set the conditions of an iterated game with the participation of LA-based agent-players (lines 1–4), a pregame (line 5), and the iterated game when players tend to achieve a Nash equilibrium corresponding to a solution of the coverage problem (lines 6–12).

First, a WSN instance of the coverage problem must be converted into a WSN graph (line 1), and next, the nodes of this graph (line 2) are to be associated with the LA-based agents of a multi-agent system. The agents are further equipped with a subset of rules from the set of five rules, and a range of changing of a parameter *k* used by rules kD, kC, and kDC is set (line 3). Next, sensor battery states are activated (line 4) with a predefined probability. To be able to start the iterated game, an initial content of LA memories must be created by performing *h* single games (line 5) when each player randomly selects a rule (from the allowed subset of rules), uses it in a game and receives some payoff, and stores the result in its memory—a pair (rule,reward).

The iterated game starts at line 6 and is continued until (line 12) a termination condition is reached—the number of allowed games is fulfilled. Each LA-based agent selects their rule (line 7) and sends this information to their neighbors, receiving from them similar information. An agent uses knowledge about the battery states of their neighbors to make a decision (line 8) concerning the modification of their battery state. Agents calculate (line 9) the values of their payoffs and spread these among player-neighbor information about new battery states. Next (line 10), they remove from their memories the oldest records with pairs (rule,reward) and store the current pairs (rule,reward). At this moment, a single game is completed, but additionally, some statistic is calculated, including the values of the two main global performance characteristics (not known by players)—a value of the coverage *q* and the number of sensors turned on n_on (line 11).

**Figure 6:**
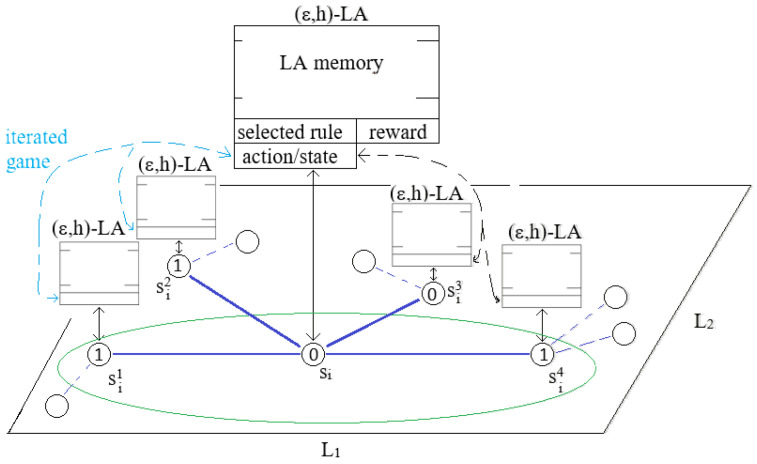
Architecture of (ϵ,h)-learning automata-based system to solve the coverage problem.

Figure 6 shows the architecture of the (ϵ,h)-LA-based system for solving the coverage problem in WSNs. One can see a part of the WSN graph with a node $s_{i}$ and its neighbors. A LA-based agent is attached to each node of the WSN graph. Each agent has its own memory of length *h* and a currently selected rule (see blocks presented in Figure 6). Details of the composition of these blocks are presented in Figure 4, and Figure 5 shows the example. Currently, the selected rule is used to change the state of a sensor controlled by the LA-based agent. The LA-based agent informs their neighbors about the new state of their sensors and receives information about the neighbor-sensor states that will be used in the next game. They then calculates their payoff using the payoff function from Table 1 and stores the pair (selected rule, reward) in their memory, deleting the oldest pair from memory. Note that a decision concerning the next LA-based agent action is taken mainly (with the probability 1 − ϵ) based on the information stored in the agent’s own memory and rarely (with the probability ϵ) by randomly selecting the action. This way of learning we will call *vertical learning*.

It is worth noting that the DE is directly represented by each player’s neighbors. Players are also assigned the DE’s duty of calculating the values of their payoff functions.

### 7.2. Learning Cellular Automata-Based Approach

In this approach, the main game-theoretical framework for searching for a solution of the coverage problem remains the same, but the main change is in the model of an agent-player. Under this approach, an agent of a multi-agent system is modeled by the learning (or adaptive) cellular automata (CA) cell. Our approach to solving the coverage optimization problem by self-organization using the SPD-like game and learning CA is summarized by Algorithm 3. While this approach was already presented in [9] in this section, we revise it to show the main differences between these two approaches.

**Figure 7:**
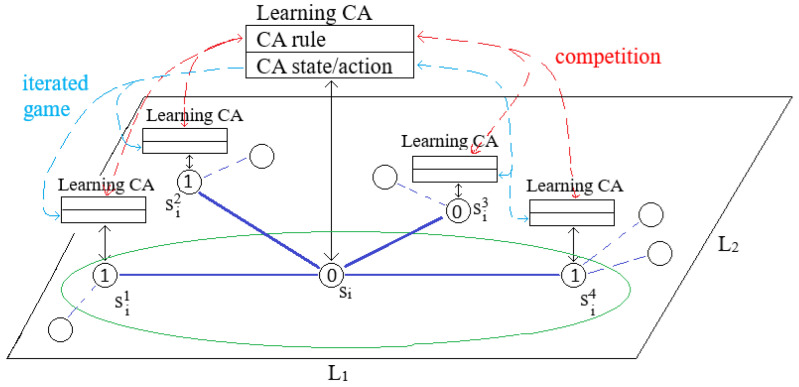
Architecture of learning cellular automata-based system to solve the coverage problem.

Figure 7 presents a general architecture of the CA-based approach to solve the coverage problem in WSN by self-organization. One can once more see a part of the WSN graph with a node $s_{i}$ and its neighbors, but in this case, a CA-based agent is attached to each node of the WSN graph. The WSN graph is considered a graph CA with an irregular structure, which is the opposite of a classical CA approach assuming a regular structure [4]. CA cells are considered nodes of the CA graph. CA cells can be in one of the following two states: 0 (corresponding to when the sensor is turned off) or 1 (corresponding to when the sensor is turned on), and the states can be changed in discrete time. Changes to CA states are performed with CA rules associated with cells. Rules, also called transition functions, are defined by the states of the local neighborhoods. Like in the previous approach, we will use of the same set of the five rules, all C, all D, kD, kC and kDC, assuming that a predefined subset of these rules is used in a given run.
**Algorithm 3** Coverage optimization by self-organization with use of learning CA  1:Convert WSN instance into WSN graph  2:Assign CA-based agent $A_{i}$ to corresponding sensors $s_{i}$—nodes of WSN graph  3:Select a subset of rules allowed to use by agents and set allowed range of changes of parameter *k*  4:With a user-predefined probability turn on the battery states of the sensors  5:Assign with a predefined probability a single rule to each $A_{i}$  6:each agent $A_{i}$ uses their current rule to change their current battery state and sends information about a new battery state and applied rule to neighbors  7:**while** termination condition is NOT TRUE **do**  8:    each agent $A_{i}$ uses their battery state as an action in the game with neighbors and calculates their payoff (according to Table 1) which is associated with their rule as a pair (rule,reward)  9:    each agent $A_{i}$ takes part in a local evolutionary competition of rules based on a local roulette wheel10:    a winner rule—a result of competition, replaces a rule of agent $A_{i}$11:    according to a predefined probability a currently assigned rule of $A_{i}$ is mutated12:   each agent-player uses their current rule to set a new battery state and sends information about a new battery state and a new rule to neighbors13:   global performance characteristics of multi-agent system—coverage *q*, and the number of sensors turned on n_on are observed14:**end while**

Not that in contrast to the LA, classical CA do not have memory, and rules assigned to CA cells cannot change over time; consequently, the system cannot learn or adapt. To transform this classical CA into an adaptive system, we transform it into a second-order CA [9,10], where the rules assigned to CA cells can change during runtime. This is achieved by introducing the following two mechanisms: a local evolutionary competition of rules and the mutation of the rules.

A local evolutionary competition of rules is based on a roulette wheel mechanism used in evolutionary computation, particularly in genetic algorithms (see, e.g., [24]), where a global operator is working on a whole population of individuals of the genetic algorithm. A local evolutionary competition in the CA approach assumes that each agent organizes competition locally (see Figure 7), i.e., an agent corresponding to the node $s_{i}$ competes with their neighbors. After a single game of CA-based players, each player knows the value of their reward, which is associated with the currently attached rule. They also know the pairs of (rule,reward) of their neighbors. They create a local roulette wheel (see, e.g., [24]) based on the values of their reward and the rewards of their neighbors. The probabilities of surviving the rules associated with rewards are calculated using these rewards. A winner rule is selected as the result of a single roulette run. If a winner rule is different than a rule currently used by a considered agent, then, their rule is replaced by the winner rule. In the opposite case, the previous rule for the considered player has not been changed. In such a way, weaker rules (i.e., rules with lower rewards) will be replaced by stronger ones during evolutionary learning. With a user-predefined probability, a rule can be subject to mutation. These new (or old) rules are further used to change the state of the corresponding CA cells, i.e., to turn on/off their batteries.

The process of learning in the CA-based approach is different from the one we can observe in the LA-based approach. This is not only based on the information stored by the agents but also on spatial information about the performance of rules belonging to agents in a neighborhood. The crucial learning mechanism in this case is a local competition mechanism. We will call this way of learning *horizontal learning*.

## 8. Nash Equilibria and Global Solutions

As already noted, our approach based on self-organization differs significantly from the classical approach based on optimization. In our approach, we replace the problem of global optimization (see Equation (2)) with the problem of reaching a Nash equilibrium by the agent-players participating in an iterated game. The level of self-organization, which we also call the level of collective behavior, is measured by the value of the ATP (see Equation (3)) at the NE, which is not known for the players, when the agent-players act in such a way so as to maximize their own payoffs.

The ATP is closely related to two other global characteristics of the system represented by parameters n_on (also unknown to the players) and $q_{r}$. The payoff function presented in Table 1 was designed in such a way as to express global parameters n_on and $q_{r}$ as the local goals of the players. A correctly designed game should link the global optimization criterion represented by Equation (2), in some way, with a value of the ATP at the NE. Linking depends on both the designed payoff function and the values of the payoff function parameters used in a game. While we have shown the correctness of designing the payoff function in [9], in this section, we focus on showing the relationships between the global solutions offered by Equation (2) with distributed solutions based on the self-organization corresponding to the ATP (Equation (3)) at the NE, which depend on the values of the parameters of the payoff function.

Due to the exponential increase in the number of solutions, an analysis of the relations between NE and the global optimization criterion can be performed only for small instances of the coverage problem. Therefore, in this study, we will use instances of WSN 5 and WSN 8, shown in Figure 1, under the assumption that $R_{s}=35$ m. WSN interaction graphs of these two instances are shown in Figure 2. For the analysis, the payoff function with the settings a=0.2,b=1.2,c=0.6,d=1.0 will be used, where $q_{r}=0.8$.

**Figure 8:**
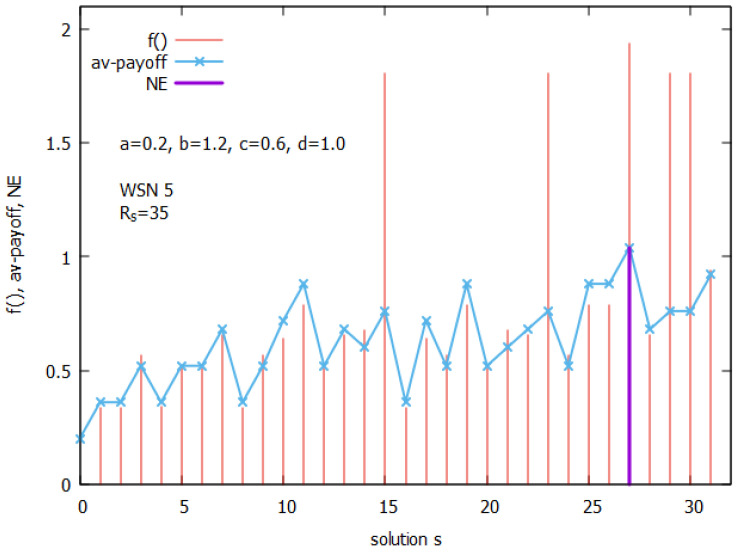
Landscape of the global criterion function f() and en external criterion ATP for WSN 5.

**Figure 9:**
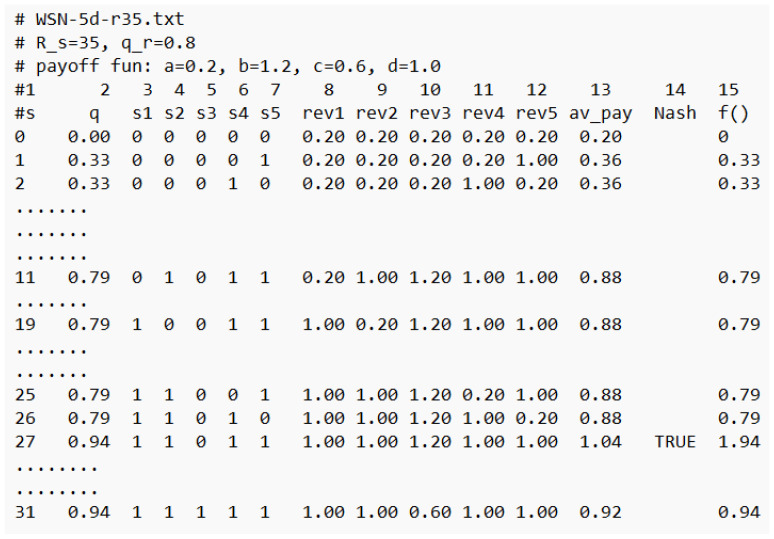
Nash equilibrium for WSN 5.

Figure 8 presents the landscapes of both functions as follows: the global function $f(q,n\text{\_}on,q_{r})$ (see Equation (2)) and the ATP (see Equation (3)) for WSN 5, and Figure 9 shows some details of the computations related to WSN 5. The space of the solutions *s* of the coverage problem consists of 32 solutions (see col. 1 of Figure 9). Figure 8 presents the values of f() (in red) and the ATP (in blue) for all solutions. One can observe that both functions indicate $s_{27}=\left(1,1,0,1,1\right)$ is an optimal solution, with corresponding values $f(s_{27})=1.94$ (see col. 15) and an ATP$(s_{27})=1.04$ (see col. 13), respectively. The solution shows that the number of sensors turned ON is equal to 4, with the corresponding value of q=0.94 (see col. 2).

Further analysis of the ATP presented in Figure 9 shows that this solution is the NE. Indeed, we can see that, e.g., player $A_{1}$ receives (col 8, rew1), at NE, a payoff equal to 1.0, but if they change the state of their battery, then, their payoff drops to the value 0.20 (see $s_{11}$, col. 8). Similar drops in payoffs can be observed for the remaining agent-players, so it does not make sense for any of them to change the state of their battery alone, which proves that $s_{27}$ is the NE, and at the same time, this is the *maximal price point* (MPP). This is a unique NE for WSN 5, and it is marked in violet in Figure 8.

**Figure 10:**
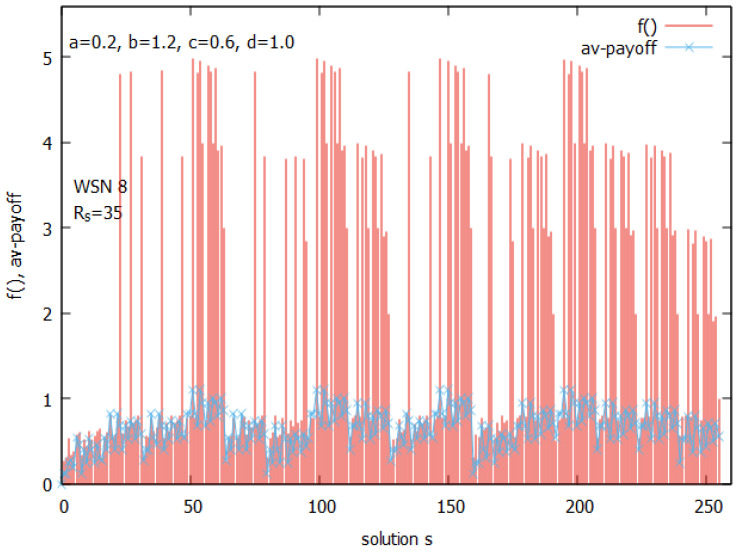
Landscape of the global criterion function f() and the external criterion ATP for WSN 8.

**Figure 11:**
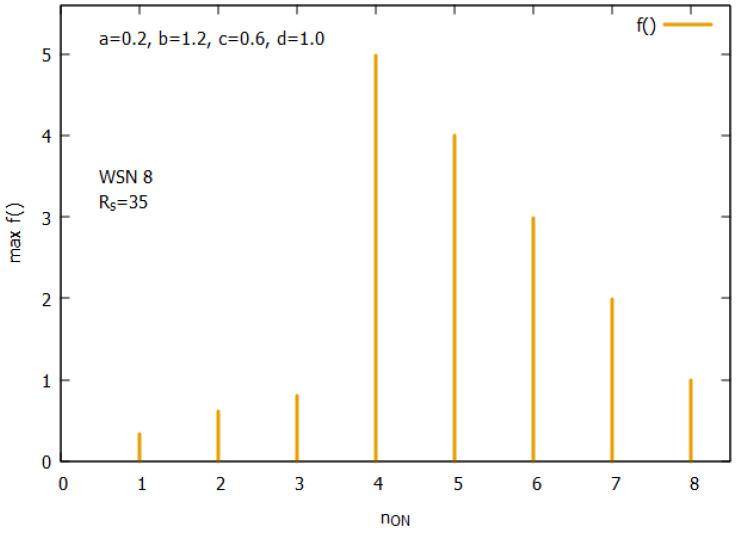
Maximal values of function f() for different values of sensors turned on for WSN 8.

**Figure 12:**
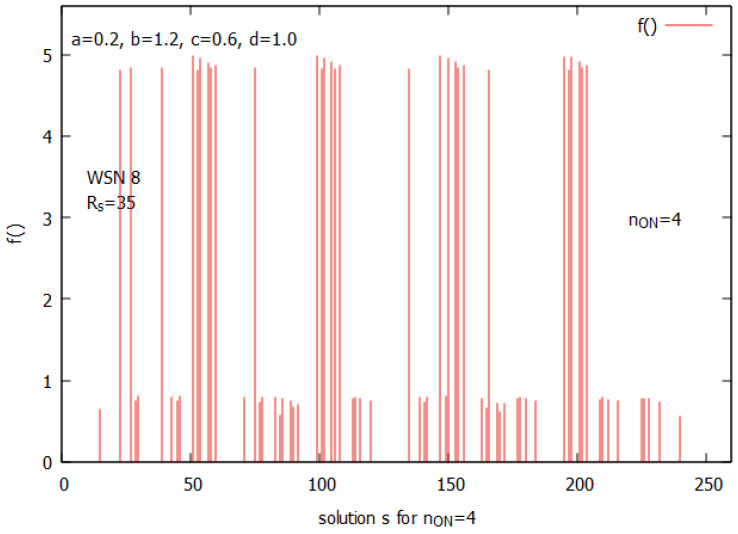
All solutions for n_on=4 for WSN 8.

Let us analyze the case of WSN 8 from Figure 2b. Figure 10 presents the landscapes of both functions f() and the ATP for WSN 8. The space of solutions *s* for this instance is much larger and consists of 256 solutions. A closer analysis of all values of f() shows (see Figure 11) that the function has its optimum for n_on=4. The figure clearly shows how function f() behaves. Its values are linearly growing with a decrease of n_on and suddenly drop after reaching an optimal value of n_on. Figure 12 presents all potential solutions for n_on=4. Twenty-eight of these solutions meet the requirement $q\geq q_{r}=0.8$ with values 4.80≤f()≤4.98, eight of them have f()≥4.95, and two of them $(s_{51}$, and $s_{147}$) represent global optima with f() = 4.98 and a corresponding value q=0.98.

**Figure 13:**
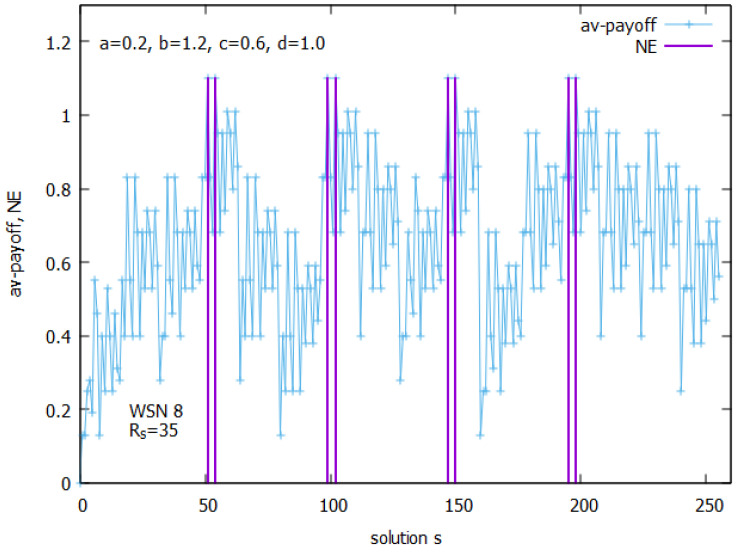
Landscape of the ATP and Nash equilibria for WSN 8.

Figure 13 presents a landscape of the ATP for WSN 8. An analysis of the NE points in the space of solutions performed similarly, as presented in Figure 9, which shows that there exist 8 NE points in the game, and these points are the same as the top eight solutions indicated by f(). These solutions are shown in Figure 13 (see in violet). They all have the same value, ATP = 1.10, and all are MPPs.

## 9. Experimental Results

Several simulation experiments have been conducted to learn the performance of the proposed methodology based on the application of LA-based agents. One of the goals was also to provide some comparison of both types of agents, i.e., LA-based agents and the recently proposed [9] CA-based agents, under the use of a common game-theoretical framework. In the experiments, we used a number of WSN instances. These are deterministic instances WSN 5, WSN 45, WSN 125, and a random instance WSN 100, where the locations of sensors are randomly selected. To be able to compare results, for most of instances, we used the same set of templates for visualization. We used the game payoff settings a=0.2,b=1.2,c=0.6,d=1.0. In all experiments, it was assumed that the requested value of coverage is $q_{r}=0.8$, and this was found by a genetic algorithm (GA) (not shown here), corresponding to a minimal number of sensors to be turned on equal to n_on=4 for all considered WSN instances. As a fitness function for the GA, Equation (2) from Section 2 was used.

In experiments where the rules using the parameter *k* are applied, we used two options to control the values of *k*. Generally, the parameter *k* can take integer values from the range 0–maxk. The first option, called *k-option* 1 assumes that *max k* is allowed to change in the range *max k = number of neighbors of the considered agent*. The second option (k−option2) assumes that *max k* is a user-predefined maximal allowed value of *k*, which is the same for all agents. In LA-based experiments, the *option* 1 is always used. In CA-based experiments, both *option* 1 and the *option* 2 are used.

Rules (strategies) can also be a subject of random changes, called mutations, which happen with a user-predefined probability pstratmut. Mutations of rules *all C* and *all D* are conducted in such a way that rule *all C* is changed to rule *all D*, and similarly, *all D* is changed into *all C*. Mutations of rules with the parameter *k* are conducted in such a way that a value of *k* is decreased or increased by 1 with a probability 0.5. Decreasing or increasing rules may be performed only within the allowed range.

### 9.1. Setting Values of Parameters h and ϵ of the LA

The purpose of the first set of experiments was to find the best values of parameters *h* and ϵ of the LA providing the optimal solution of the coverage problem. The issue of retrieving an optimal pair (q(s),n_on(s)) under all possible combinations *s* of sensors being turned on or off was discussed in Section 2, from the perspective of a single run of the algorithm. According to that discussion, it is necessary to find the minimal value of n_on that provides the maximal value of *q*, meeting the requirement $q\geq q_{r}$.

**Figure 14:**
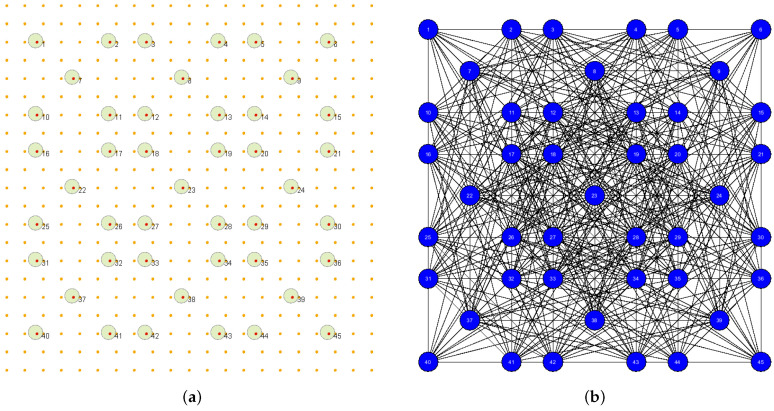
WSN 45: (**a**) sensors localization, (**b**) interaction graph ($R_{s}=30$ m).

**Figure 15:**
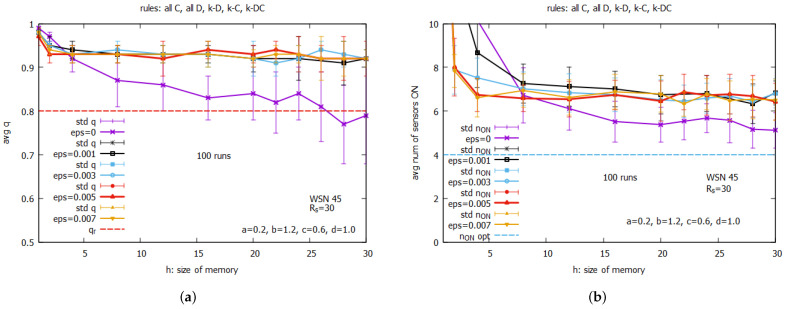
Searching for optimal values of LA parameters: (**a**) coverage *q* as a function of *h* and ϵ, (**b**) the number of sensors $n_{ON}$ as a function of *h* and ϵ.

The experiments were conducted with the instance WSN 45, which consists of 45 sensors with a sensing range $R_{s}=30\;m$ (see Figure 14), using of the whole set of five rules. The results of the experiments were averaged over 100 runs and are presented in Figure 15. Figure 15a and Figure 15b present the changes in the averaged values of the coverage *q* and a number of sensors turned on n_on, respectively, as a function of parameters *h* and ϵ. Because we are dealing with averaged values, we should interpret the experimental results not only from the perspective of the averaged values *q* and n_on but also by considering the values of the standard deviations.

One can see (Figure 15a) that practically for all values of ϵ, the values of *q* exceed the value of $q_{r}$, except for the case when ϵ=0 (in violet), when this request is not fulfilled for h>25. At first glance, the case when ϵ=0 and h<25 seems to be a good candidate for establishing an optimal solution because we can see (Figure 15b) that the values of n_on are the lowest, which means this is equivalent to the minimal spending energy of the sensor batteries. However, when we look at Figure 15a, we can observe the relatively large values of the standard deviations of *q*, which signal that in single runs, the values of *q* can sometimes be below $q_{r}$. This is why we exclude this case from further considerations.

The similar analysis of cases with other values of ϵ brings us to the conclusion that the curve corresponding to ϵ=0.05 (in red) best meets our expectations. We can see that while values of *q* for ϵ=0.05 are close to the ones corresponding to other values of ϵ, the value h=8 for ϵ=0.05 provides a minimal value of n_on. Therefore, the pair ϵ=0.05 and h=8 are used in further experiments with the LA-based approach.

In the case of specific applications of the considered LA-based algorithm and specific requirements concerning the acceptable level of the relaxation of the degree *q* dropping below $q_{r}$, the search for values of parameters *q* and n_on should be based on analyzing single runs of the algorithm.

### 9.2. LA-Based Approach: The Instance WSN 5

The purpose of this set of experiments was to gain some insights into the work of the self-organizing algorithm when a small instance of the problem WSN 5 is used. The experiments have been conducted under the assumption that $R_{s}=35$ m is used. The WSN interaction graph of the multi-agent system is presented in Figure 2a, and a theoretical analysis of the game is presented in Section 8. Experiments have been conducted under the assumption that the whole set of five rules is used.

**Figure 16:**
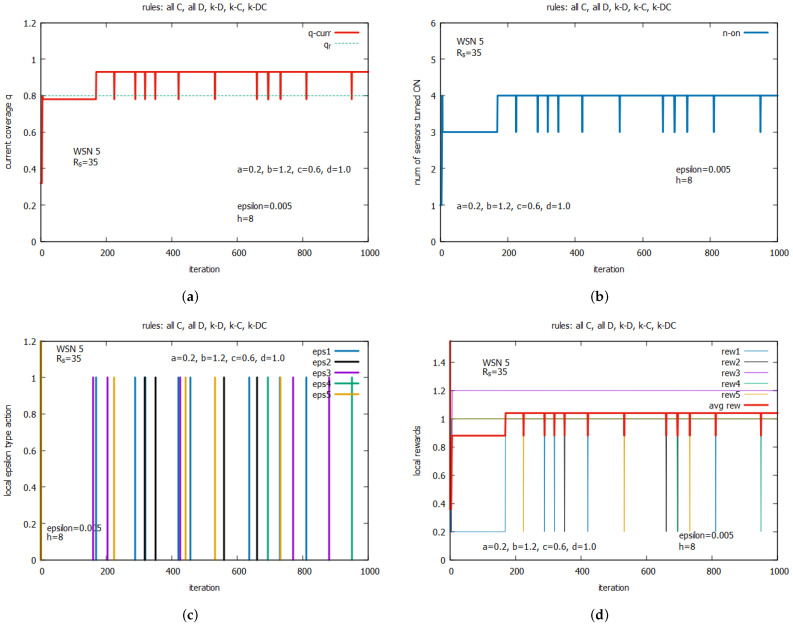
Single run for WSN 5: (**a**) coverage *q*, (**b**) number of sensors $n_{ON}$, (**c**) moments of taking actions by agents caused by the ϵ alternative of the LA algorithm, and (**d**) local rewards of agents.

**Figure 17:**
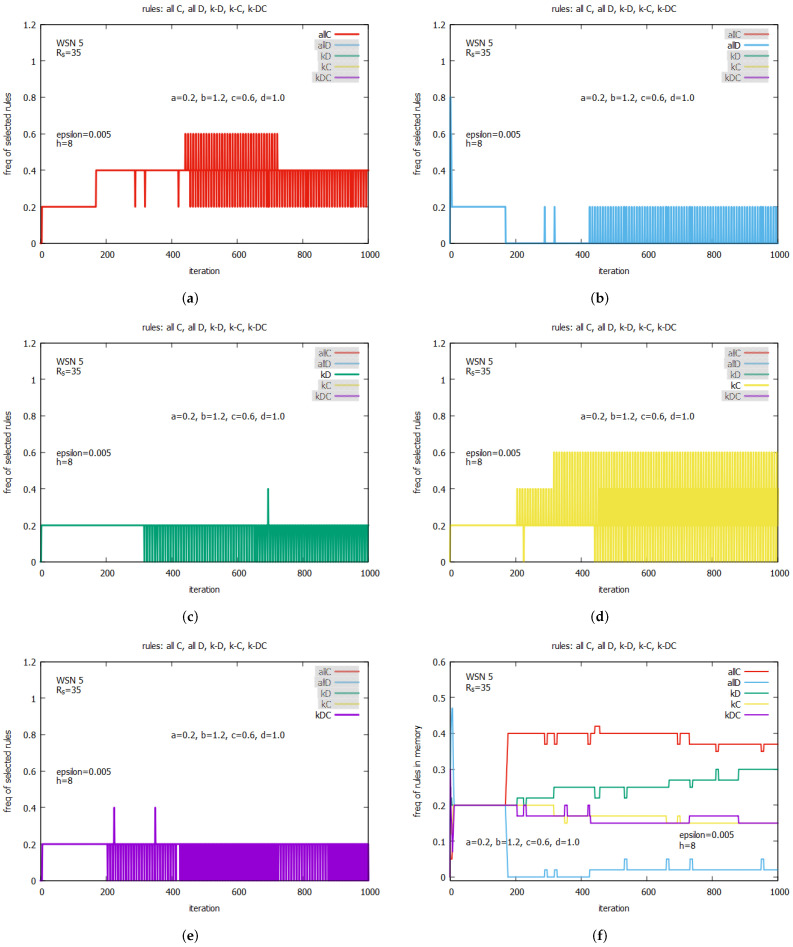
Single run for WSN 5: (**a**) frequency of rule *all C*, (**b**) frequency of rule *all D*, (**c**) frequency of rule kD, (**d**) frequency of rule kC, (**e**) frequency of rule kDC, (**f**) fractions of rules stored in LA memories.

Figure 16 and Figure 17 present the results of a single run of the algorithm. Figure 16a and Figure 16b show the changes in the global parameters *q* and n_on, respectively. One can see that a suboptimal solution characterized by q=0.78 and n_on=3 was reached very quickly at iteration 7, and finally, this was moved into an optimal solution in iteration 169, characterized by q=0.94 and n_on=4. This solution was expected from the analysis provided in Section 8. This remains stable until the end of the iterated game because it corresponds to a unique NE in this game.

Figure 16c shows moments in time when some agents work out their actions using the ϵ alternative of the LA algorithm. The first use of the ϵ alternative happens at iter = 161 (see impulse line in violet) by agent 3, but it does not result in any changes in the system. The second use of the ϵ alternative happens at iter = 169 (see impulse line in blue) by agent 1 and results in the shift from the suboptimal to the optimal solution. Indeed, agent 1 changed their *all D* rule, which was used up until that point, to rule *all C*, which is the result of a change in the state of sensor 1 from 0 to 1.

Figure 16d shows how the rewards of agents change in the game. One can see that before moving from the suboptimal solution to the optimal one, the reward for player 1 was equal to 0.2 (see the line in blue), the reward for player 3 was equal to 1.2 (see the line in violet), and the rewards for the remaining three players were equal to 1.0. When player 1, at iter = 169, changes the state of their battery to 1, the suboptimal solution became the optimal one, as we already noted, and this moved their personal reward to 1.0, while the rewards for remaining players did not change. The optimal solution reached by the players is characterized by the highest average payoff (see the line in red) of the game (ATP) equal to 1.04, which corresponds to a specific NE called the MPP. We can see that the solution is stable for the remaining games, despite attempts to change the course of the game, mostly caused by the ϵ alternative of the LA.

Figure 17 gives some insight into the process of managing rules of LA which collectively influence the global performance of the system. Figure 17a–e show how the frequencies of rules selected by agents change in time. Until iter = 169, all five rules are used with the same frequency, equal to 0.2, but later on, we can observe a complex dynamic, where different rules are used. In the period between iter = 170 and iter = 252, rule *all C* (in red) dominates, and it is used with a frequency of 0.4, the frequency of rule *all D* (in blue) is equal to 0, and the frequencies of the remaining rules *kD* (in green), *kC* (in orange), and *kC* (in violet) are equal to 0.2.

In the next period, between iterations 253 and 309, both rules *all C* and *kC* have frequencies equal to 0.4, *kD* has the frequency equal to 0.2, and *all D* has a frequency equal to 0. At iter = 317, we can observe that *kC* reaches the highest frequency, which is equal to 0.6, and *all C* still has the same frequency, which is equal to 0.4. In the next iterations until iter = 445, the frequencies of the rules return to the pattern observed in the period 170–252, but the pattern observed at iter = 317 returns periodically. At iter = 450, *all C* reaches the highest frequency equal to 0.6, *all D* becomes active with a frequency of 0.2 and *kD* with a frequency of 0.2. In further iterations, we can see that two rules, *all C* and *kC*, dominate, periodically reaching, albeit in different phases, frequency values equal to 0.6, before moving to frequency 0.4, then, 0.2, and returning back to 0.6.

Figure 17f gives some insights into the contents of the whole set of the LA memories consisting of 5∗(h=8)=40 units. One can see that, until iter = 169, all rules are equally distributed with a frequency equal to around 0.2. In further iterations, we can observe the process of significant changes in LA memory structure. Rule *all C* becomes the dominating rule, occupying around 40% of the LA memory structure, and *all D* occupies only around 0.05 of this structure. We can observe an increase in the number of *kD* rules, finally reaching a percentage of around 30% in terms of the entire rule population, and at the same time, the populations of *kC* and *kDC* are decrease to around 0.15%.

**Figure 18:**
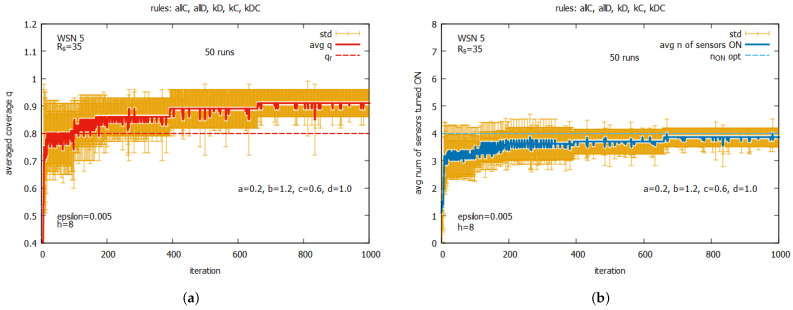
WSN 5: (**a**) averaged value of coverage *q*, (**b**) averaged value of the number n_on of sensors turned on.

Figure 18 presents the main averaged results over 50 runs concerning of WSN 5. One can see that the requested coverage $q_{r}$ is reached, on average, after around 100 iterations (see Figure 18a), but the process of searching for a solution is accompanied by a noticeable standard deviation. Improving the average values of *q* and n_on (see Figure 18b) can be observed in the next iterations, with decreases in the values of the standard deviation.

### 9.3. LA-Based Approach: The Instance WSN 45

**Figure 19:**
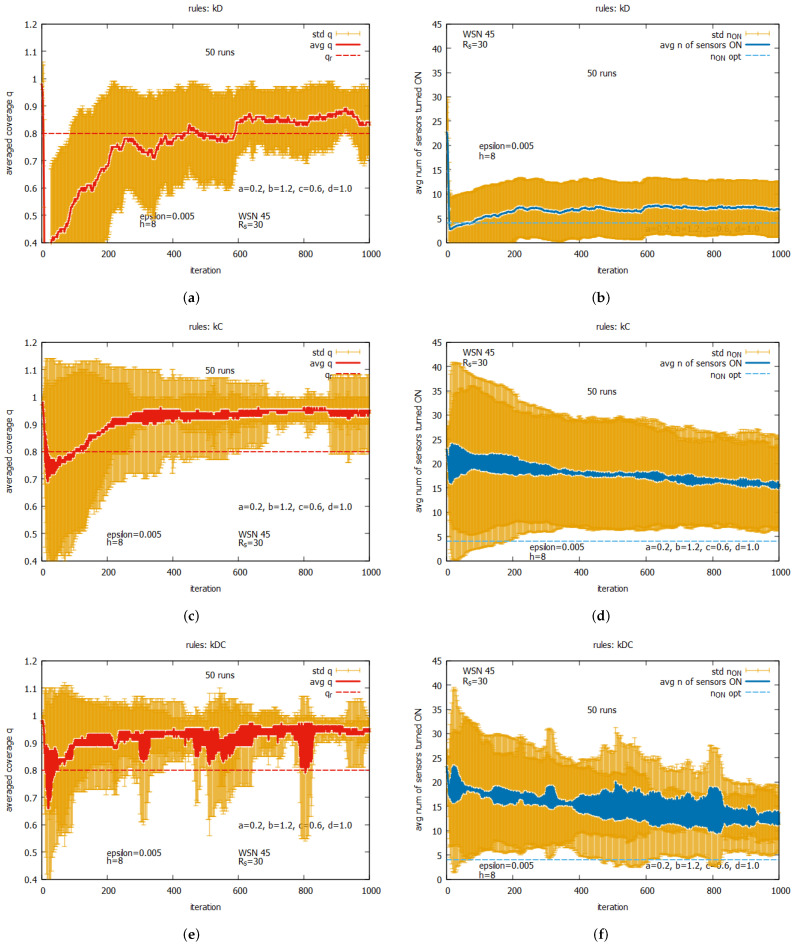
WSN 45: averaged value of (**a**) coverage *q* for rule kD, (**b**) the number n_on of sensors turned on for rule kD, (**c**) coverage *q* for rule kC, (**d**) the number n_on of sensors turned on for rule kC, (**e**) coverage *q* for rule kDC, (**f**) the number n_on of sensors turned on for rule kDC.

**Figure 20:**
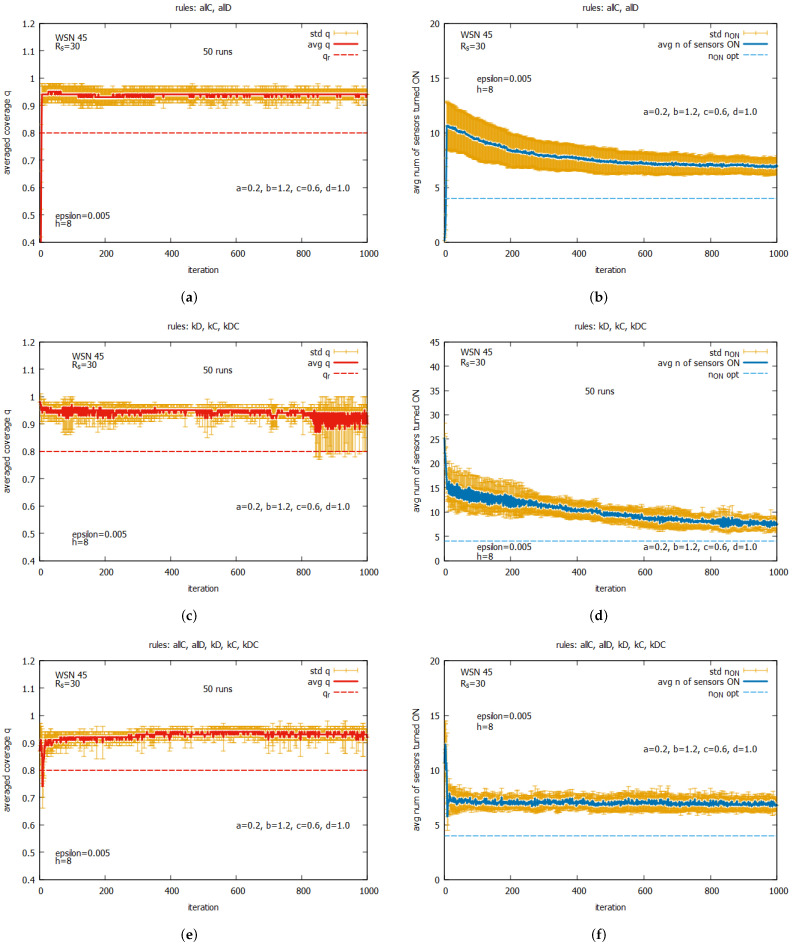
WSN 45: averaged value of (**a**) coverage *q* for rules {all C,all D}; (**b**) the number n_on of sensors turned on for rules {all C,all D}; (**c**) coverage *q* for rules {kD,kC,kDC}; (**d**) the number n_on of sensors turned on for rules {kD,kC,kDC}; (**e**) coverage *q* for rules {all C,all D,kD,kC,kDC}; (**f**) the number n_on of sensors turned on for rules {all C,all D,kD,kC,kDC}; (**d**) the number n_on.

**Figure 21:**
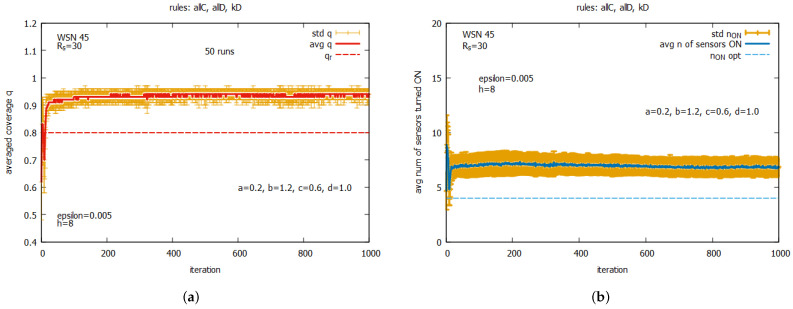

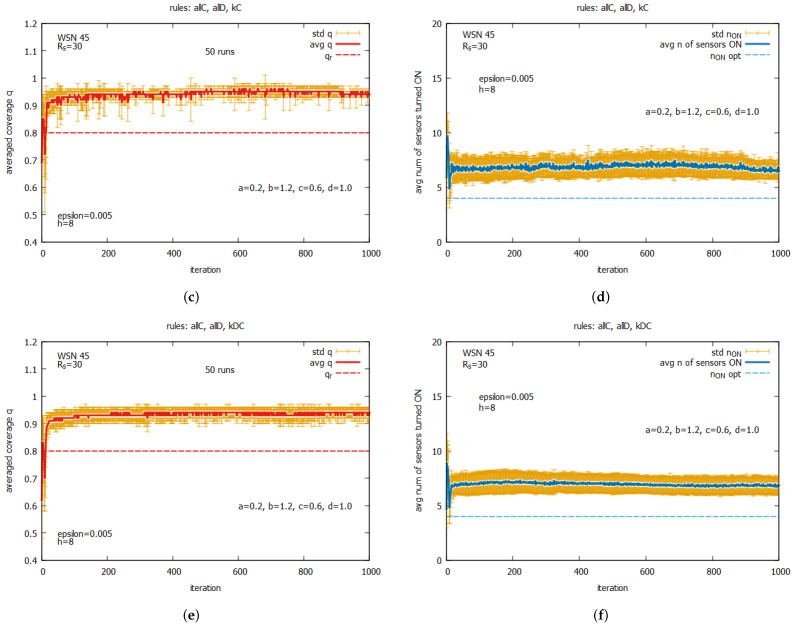
WSN 45: averaged value of (**a**) coverage *q* for rules {all C,all D,kD}; (**b**) the number n_on of sensors turned on for rules {all C,all D,kD}; (**c**) coverage *q* for rules {all C,all D,kC}; (**d**) the number n_on of sensors turned on for rules {all C,all D,kC}; (**e**) coverage *q* for rules {all C,all D,kDC}; (**f**) the number n_on of sensors turned on for rules {all C,all D,kDC}.

The instance WSN 5 is useful for verifying game-theoretic concepts, but it is too small to judge the general performance of the proposed approach. Therefore, in this subsection, we return to a more realistic instance of the problem, called WSN 45 (Figure 14), which we have already presented. The main goal of the experiments was to find out how different subsets of rules used by LA-based agents work collectively towards achieving the requested goals described by *q* and n_on. The averaged results of the experiments are presented at Figure 19, Figure 20 and Figure 21.

Figure 19 presents the averaged values of *q* and n_on for the following individual rules: kD, kC, and kDC. The averaged values of *q* are shown in Figure 19a for rule kD, Figure 19c for kC, and Figure 19e for kDC. We can observe the exact averaged values of *q* (marked as a red line), the standard deviation of *q* (marked in orange), and the requested *q* (marked as a dashed red line). The averaged values of *n* sensors turned ON are presented in Figure 19b for rule kD, Figure 19d for kC, and Figure 19f for kDC. Here, we can see the exact averaged values of n_on (marked as a blue line), the standard deviation of n_on (marked in orange), and the optimal value of n_on (marked as a dashed blue line). One can see that all individual rules can reach a level of *q* exceeding the requested value of $q_{r}$. This occurs with different speeds of converging *q* and varying final numbers of sensors turned on.The fastest convergence of *q* is offered by rule kDC (see Figure 19e,f), with the average number of sensors turned on being around 14. A slightly longer convergence can be observed for rule kC (see Figure 19c,d), with the average number of sensors turned on being around 16. The longest time of convergence can be observed for rule kD (see Figure 19a,b), with the average number of sensors turned on being around five. While a large standard deviation is observed for all these rules, it is the lowest for rule kD. The general conclusion from this set of experiments is that these individual rules cannot be considered as solvers on their own, but they may serve as effective building blocks when used together with other rules. In the next set of experiments, we will assess the performance of other potential building blocks.

Figure 20 shows the averaged values of *q* and n_on for rules *all C* and *all D* (a,b) working together; for rules kD, kC, and kDC (c,d) working together; and for all five rules (e,f) working together. One can observe that for all of these rule combinations, convergence to higher values *q* is reached very fast, and the standard deviations are noticeably lower than the previous experiment. The best performance is demonstrated by the system which uses five rules when *q* achieves the average value equal to around 0.9, with the associated cost of requiring around seven sensors to be turned on. The next experiment (see Figure 21) presents the performance of the system for three rule combinations as follows: *all C*, *all D*, and kD (a,b); *all C*, *all D*, and kC (c,d); and *all C, all D*, and kDC (e,f). One can observe a further decrease in the standard deviation when *q* and n_on are reached.

**Figure 22:**
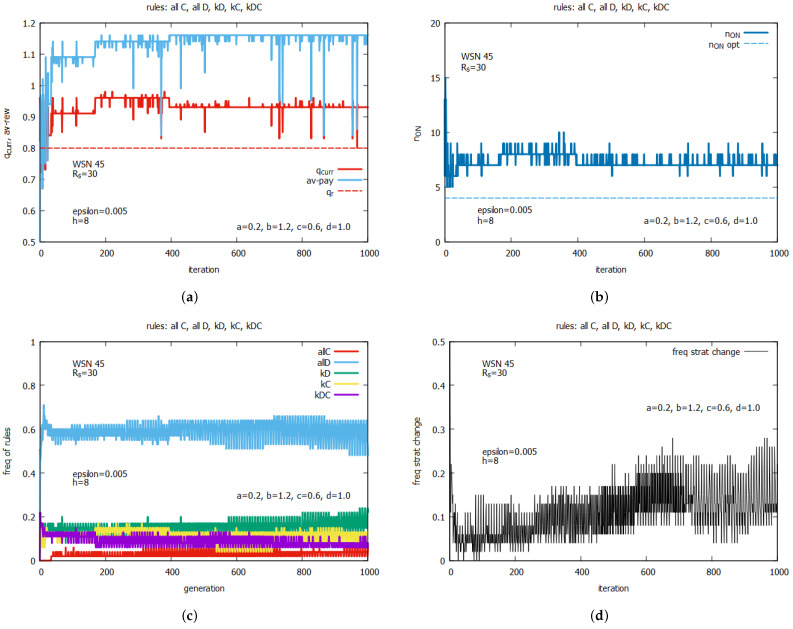

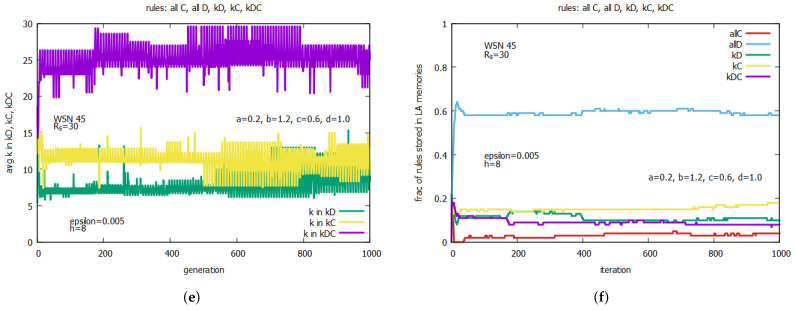
WSN 45 (a single run): averaged value of (**a**) coverage *q*, (**b**) the number n_on of sensors turned, (**c**) a frequency of rules applied by LA-based agents; (**d**) a frequency of changing rules, (**e**) average values of *k* for the {kD,kC,kDC}rules; (**f**) fractions of rules stored in LA memories.

Figure 22 is a concluding experiment conducted with WSN 45, which presents the results of a single run of the algorithm using the whole set of five rules. The purpose of this experiment is to provide some insight into the work of the algorithm. Figure 22a shows the current values of *q* and the other global parameters of the system (not known for agents)—the average total reward av−rew corresponding to the ATP (see Equation (3)). One can notice that the value of *q* is always above the requested value $q_{r}$. Figure 22b presents the current values of n_on, which oscillate around 7. Figure 22c shows the frequency of rules selected by agents to change the sensor states. One can see that rule *all D* (in blue) is the most frequently selected rule by LA-based agents, while rule *all C* (in red) is the least selected rule. Rule kD (in green) is selected with a frequency equal to around 0.2. The frequencies of rules kC (in orange) and kDC (in violet) are lower than those of kD and higher than *all C*. Figure 22d shows the value of the other global parameters—the frequency of the strategies changes. One can see that the frequency of change in rules selected by agents is relatively high and equal to around 0.15. Figure 22e shows the changes in the averaged values of the parameter *k*, used by rules kD,kS, and kDC. One can see that the highest value of *k*, oscillating around 25, is related to strategy kDC (in violet). The average value of *k* of strategies kC (in orange) oscillates around 12, and in the case of kD (in green), it oscillates around 7. Figure 22f shows the fractions of the rules stored in the total LA memory. One can see that around 60% of the stored rules are *all D* rules, and the fractions of the other stored rules are below 0.2.

### 9.4. CA-Based Approach: The Instance WSN 45

In this Subsection we report some experiments using the CA-based approach to compare them with the results obtained using the LA-based approach. Let us notice that both algorithms use the same game-theoretic model.

**Figure 23:**
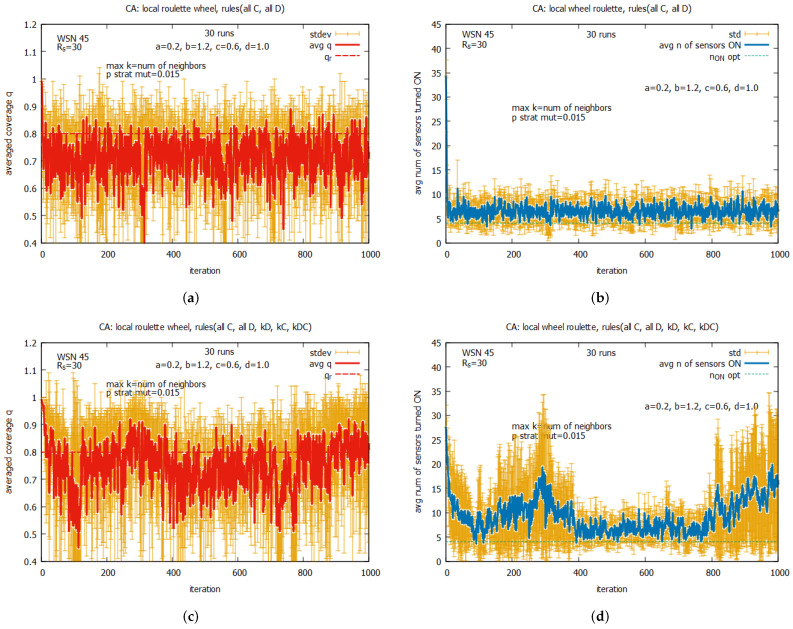
WSN 45 (CA-based approach: k-option 1). Rules all C and all D: averaged value of (**a**) coverage *q* for rules all C and all D, (**b**) the number n_on of sensors turned on for rules all C and all D, (**c**) coverage *q* for a whole set of five rules, (**d**) the number n_on of sensors turned on for a whole set of 5 rules.

Figure 23 presents results averaged over a 30-run simulation for WSN 45, using CA for the following two algorithm options: an option that uses two rules, {*all C* and *all D*} (see Figure 23a,b), and an option that uses the whole set of five rules (see Figure 23c,d). The values of *k* in this experiment were controlled by *k-option* 1, i.e., like it was in experiments with LA-based agents. The presented results show that for both considered algorithm options, we have to do with a relatively large standard deviations of coverage *q*. We can see also that the average values of *q* are mostly below the requested $q_{r}$. The algorithm option with the two rules is slightly better and more promising than the option with the five rules. It provides much more stable values for both *q* and n_on. For the above reasons, we continue experiments using *k-option* 2.

The first set of experiments was conducted with single rules, kD, kC, and kDC, working separately. The results of the experiments (not shown here) suggest that, for rules kD and kDC, the system nearly immediately convergence to a coverage q=0, and for kC, we can observe a regular oscillation between q=0 and q=1.

**Figure 24:**
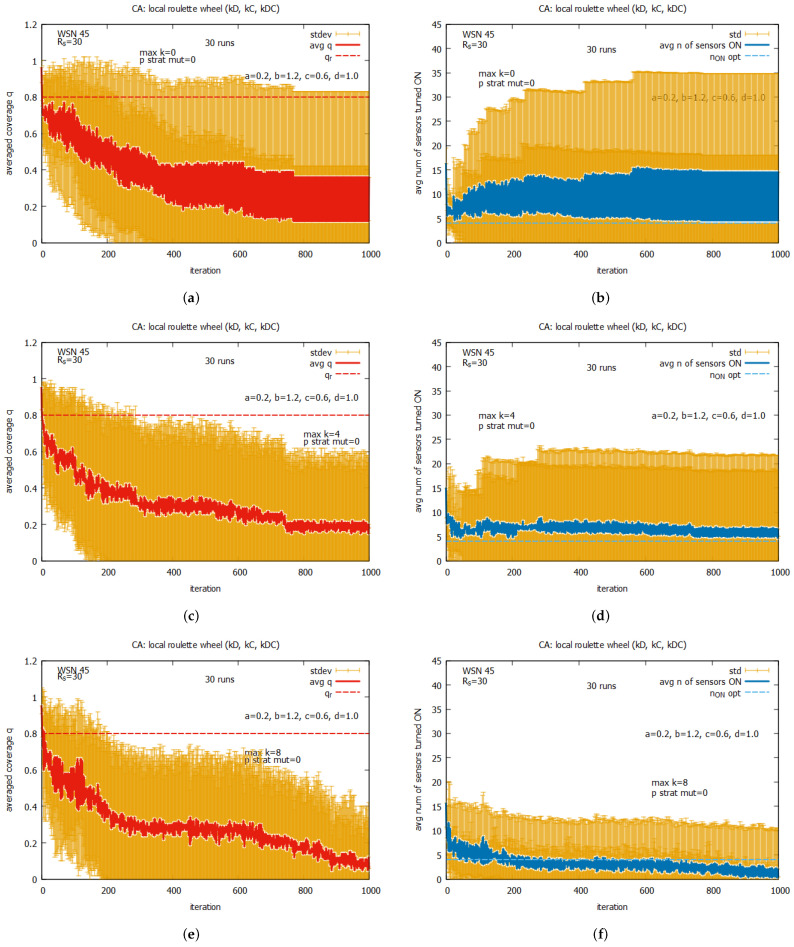
WSN 45 (cellular automata-based approach: k-option 2) Rules kD, kC, and kDC, pstratmut=0: averaged value of (**a**) coverage *q* for maxk=0, (**b**) the number n_on of sensors turned on for maxk=0, (**c**) coverage *q* for rules for maxk=4 (**d**) the number n_on of sensors turned on for maxk=4, (**e**) coverage *q* for maxk=8, (**f**) the number n_on of sensors turned on for maxk=4.

Figure 24 presents the averaged values of *q* and n_on for rules kD, kC, and kDC working collectively. We conducted experiments with values *max k* ranging between 0 and 16, under the assumption that the probability of strategy mutation (*p strat mut*) is equal to 0. We can see that, contrary to the first experiment, the system is adaptive and converges to *q*, which is equal to around 0.2; however, this is significantly below $q_{r}$. We can also notice that, despite of the high values of standard deviation, the average value of *q* and n_on become the lowest and stabilize for maxk=4 (see Figure 24c,d). This value of *max k* will be used in the remaining experiments with CA-based agents.

**Figure 25:**
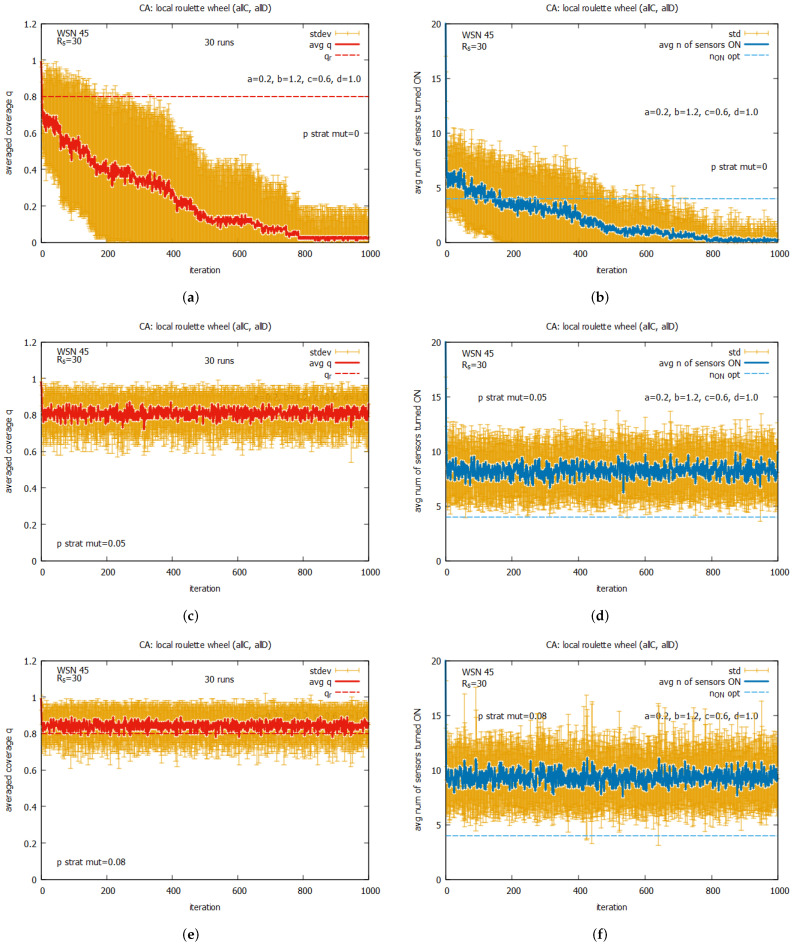
WSN 45 (cellular automata-based approach: k-option 2) Rules all C and all D: averaged value of (**a**) coverage *q* for pstratmut=0, (**b**) the number n_on of sensors turned on for pstratmut=0, (**c**) coverage *q* for pstratmut=0.05, (**d**) the number n_on of sensors turned on for pstratmut=0.05, (**e**) coverage *q* for pstratmut=0.08, (**f**) the number n_on of sensors turned on for pstratmut=0.08.

Figure 25 shows the averaged values of *q* and n_on for rules *all C* and *all D* working collectively, under the value of *max k = 8* and with different values of *p strat mut*. One can see that the convergence of the system to the optimal value of *q* can be observed only when the value pstratmut>0. The average value of *q* equal to $q_{r}$ is reached for pstratmut=0.05, with a relatively low average value of n_on (see Figure 25c,d), but if we want to reduce the risk of having values of *q* below $q_{r}$, the strategy mutation should be higher (see Figure 25e,f). We can observe some drawback of the CA-based approach in comparison with the LA-based approach—a much higher standard deviation of the final solution.

**Figure 26:**
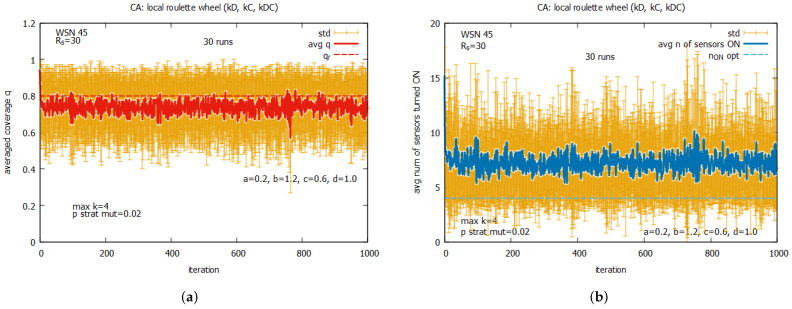

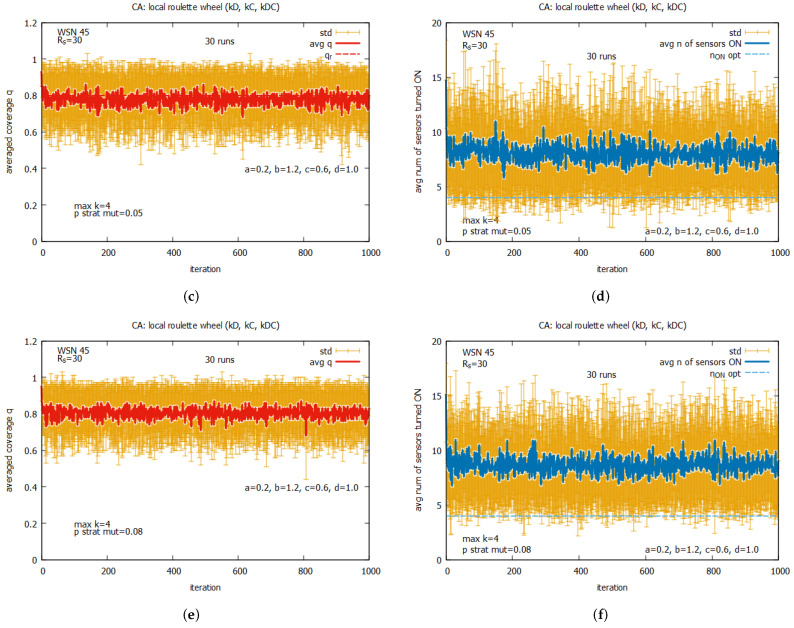
WSN 45 (cellular automata-based approach: *k*-option 2) Rules kD, kC, and kDC, maxk=4: averaged value of (**a**) coverage *q* for pstratmut=0.02, (**b**) the number n_on of sensors turned on for pstratmut=0.02, (**c**) coverage *q* for rules for pstratmut=0.05, (**d**) the number n_on of sensors turned on for pstratmut=0.05, (**e**) coverage *q* for pstratmut=0.08, (**f**) the number n_on of sensors turned on for pstratmut=0.08.

In the next experiments, we return again to the system working collectively when the set of rules kD, kC, and kDC is used. Now, the experiments are conducted under the assumption that maxk = 4 but pstratmut is changed, and Figure 26 presents selected results for some values of pstratmut. Now we can see that the system converges very quickly to *q*, which is close to $q_{r}$, but the quality of the proposed solutions depends on the values of pstratmut. For pstratmut=0.02, the average value of *q* is slightly below $q_{r}$ (see Figure 26a,b), and for pstratmut=0.05, it is around $q_{r}$ (see Figure 26c,d). Further increasing mutation pstratmut=0.08 slightly improves the average values of *q* and n_on (see Figure 26e,f), and this value will be used in further experiments with CA-based agents.

**Figure 27:**
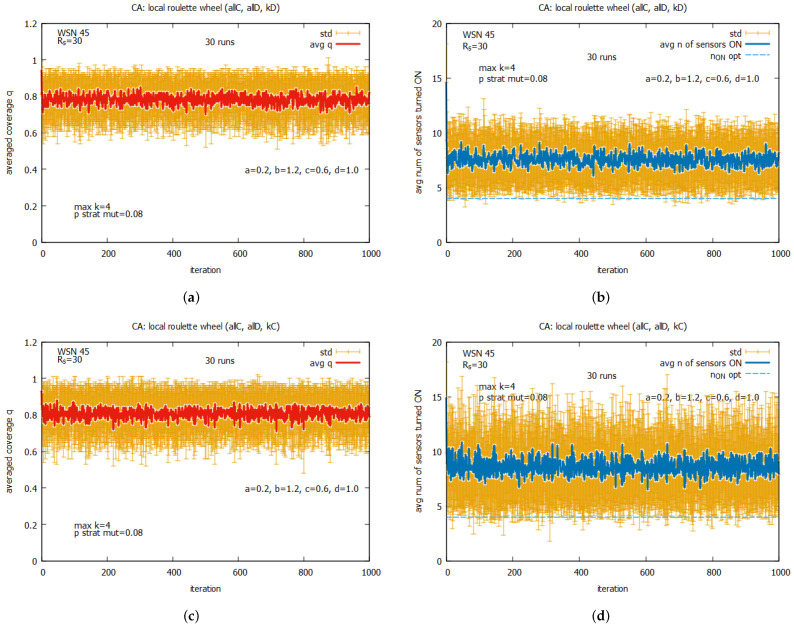

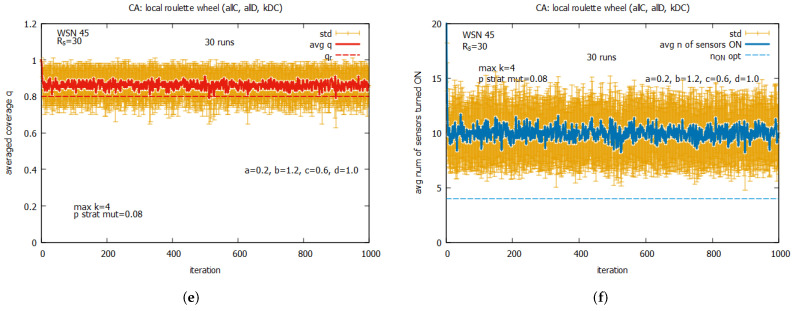
WSN 45 (cellular automata-based approach: *k*-option 2): averaged value of (**a**) coverage *q* for rules all C, all D, and kD; (**b**) the number n_on of sensors turned on for rules all C, all D, and kD; (**c**) coverage *q* for rules all C, all D, and kC; (**d**) the number n_on of sensors turned on for rules all C, all D, and kC; (**e**) coverage *q* for rules all C, all D, and kDC; (**f**) the number n_on of sensors turned on for rules all C, all D, and kD.

**Figure 28:**
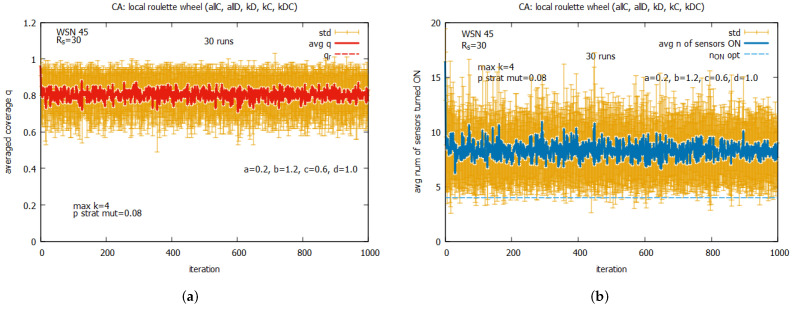
WSN 45 (cellular automata-based approach: *k*-option 2) Rules all C, all D, kD, kC and kDC: (**a**) averaged value of coverage *q*, (**b**) averaged value of the number n_on of sensors turned on.

Figure 27 presents the results of the collective behavior of two rules *all C*, *all D* working together with single rules kD (see Figure 27a,b), kC (see Figure 27c,d), and kDC (see Figure 27e,f). The experiments were conducted under maxk=4 and pstratmut=0.08. In the first two cases, we can see a solution is quickly reached, providing the average value of *q* that is equal to around the requested value of 0.8, and in the third case, the system provides a solution with an average value of *q* above $q_{r}$, which requires, however, an increase in the value of the average number of sensors turned on, equal to around 10. Figure 28 shows the results of the last experiment with CA-based agents, with use of the whole set of five rules. One can see that the results are similar to the ones presented in Figure 27a,b, which suggests that rule kD has greater influence on the performance of the system compared to rules kC and kD.

Comparing the results obtained for the LA-based agents and CA-based agents, we can observe two important differences between them. The first difference is that the LA-based approach provides values of coverage *q* much higher than the requested $q_{r}$, with relatively good costs expressed in the number of sensors turned on. The CA-based approach provides a *q* which is on the border of the requested $q_{r}$ and requires more sensors to be turned on. The second difference is that the CA-based agents approach is characterized by significantly higher standard deviations of the provided solutions compared to the standard deviations observed with LA-based systems, which may be problematic in real applications. On the basis of this comparison, we conclude that the LA-based approach significantly outperforms the CA-based approach.

**Figure 29:**
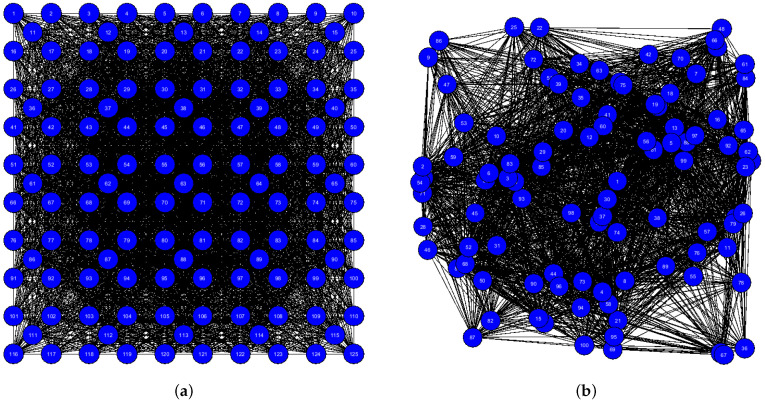
Interaction graphs for $R_{s}=30$: WSN 125 (**a**); WSN 100 rand (**b**).

In the next Subsections we continue to study LA-based self-organizing systems to see how scalable the proposed approach is. To achieve this, we will use four instances with a higher number of sensors. The first of them is WSN 125 (see Figure 29a) with 125 sensors deterministically located in the monitored area. The second one is WSN 100 (see Figure 29b) with 100 sensors randomly located in the monitored area.

### 9.5. LA-Based Approach: The Instance WSN 125

**Figure 30:**
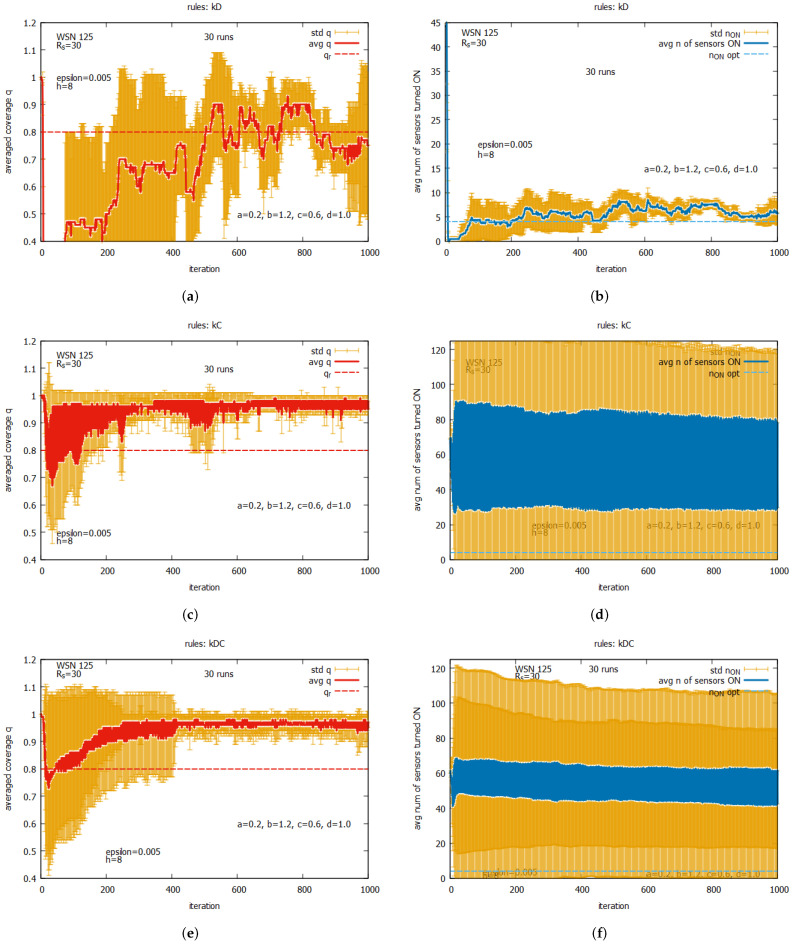
WSN 125: averaged value of (**a**) coverage *q* for rule kD, (**b**) a number n_on of sensors turned on for rule kD, (**c**) coverage *q* for rule kC, (**d**) the number n_on of sensors turned on for rule kC, (**e**) coverage *q* for rule kDC, (**f**) the number n_on of sensors turned on for rule kDC.

**Figure 31:**
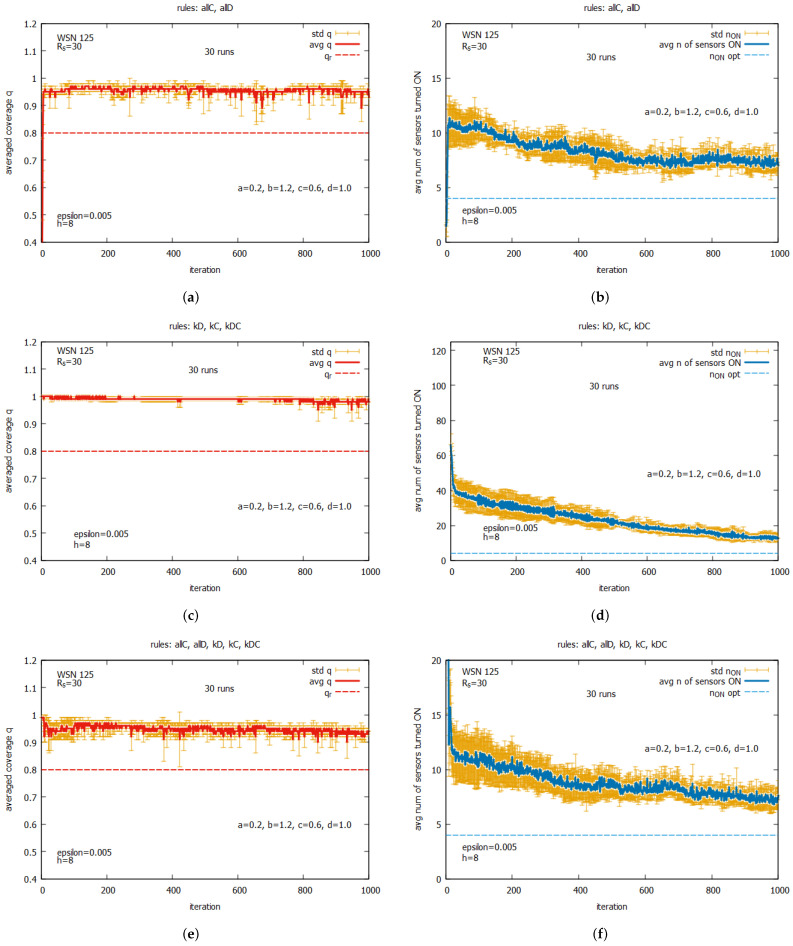
WSN 125: averaged value of (**a**) coverage *q* for rules {all C,all D}; (**b**) the number n_on of sensors turned on for rules {all C,all D}; (**c**) coverage *q* for rules {kD,kC,kDC}; (**d**) the number n_on of sensors turned on for rules {kD,kC,kDC}; (**e**) coverage *q* for rules {all C,all D,kD,kC,kDC}; (**f**) the number n_on of sensors turned on for rules {all C,all D,kD,kC,kDC}; (**d**) the number n_on.

**Figure 32:**
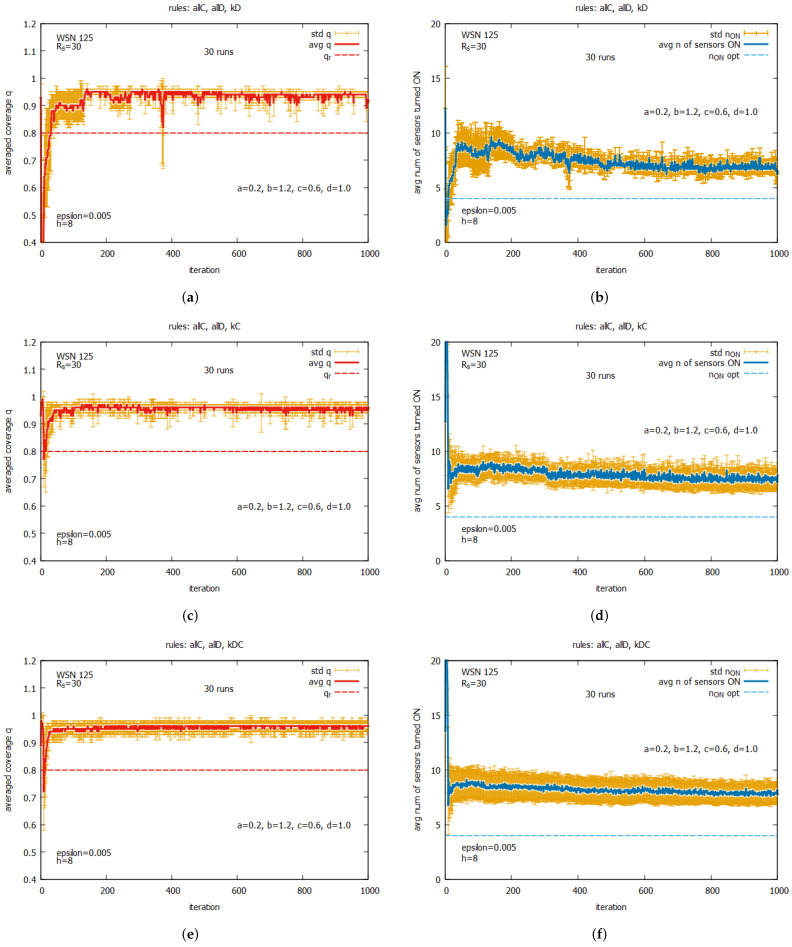
WSN 125: averaged value of (**a**) coverage *q* for rules {all C,all D,kD}; (**b**) the number n_on of sensors turned on for rules {all C,all D,kD}; (**c**) coverage *q* for rules {all C,all D,kC}; (**d**) the number n_on of sensors turned on for rules {all C,all D,kC}; (**e**) coverage *q* for rules {all C,all D,kDC}; (**f**) the number n_on of sensors turned on for rules {all C,all D,kDC}.

The averaged results of the experiments for WSN 125 are presented in Figure 30, Figure 31 and Figure 32. Figure 30 presents results assuming that only one of rules kD, kC, or kDC is used, and we may compare these results with the ones obtained for WSN 45 (see Figure 19). Comparing the behavior of kD (a,b), we can observe that the average speed of convergence of *q* is slightly longer in the case of WSN 125, and this is reached, for the first time, at the iteration around 550 (for WSN 64, at iteration around 450). Time to time drops below the requested $q_{r}$, while for WSN 45, it drops below $q_{r}$ only one time. When we compare the averaged values of n_on, we can see that in both cases, the averaged n_on is above 5 but the standard deviation for WSN 125 is much lower than for WSN 45. For rules kC and kDC, we can observe that the averaged value of *q* is close to 1 (similar to what it was in the case of WSN 45) and it requires turning on around 40% of the sensors. However, in the case of WSN 125, we can observe much higher values of the averaged number of sensors turned on and much higher standard deviations.

Figure 31 presents the results for the set of rules *all C* and *all D* (see Figure 31a,b). For the set kD, kC, and kDC, see Figure 31c,d), and for the whole set of five rules (see Figure 31e,f). Again, we can compare these results with the ones obtained for WSN 45 (see Figure 20). One can see that the results for both instances are similar. The best results are obtained for the set of five rules, and the set of rules *all C* and *all D*, when the averaged value of n_on is around 7, provide a level of coverage equal to 0.95. The set kD, kC, and kDC offers solutions that provide a value of *q* equal to 1, but this requires, on average, around 12 sensors to be turned on. While for WSN 45, the system using a set of five rules achieves a solution very quickly, in the case of WSN 145, we can observe that the number of sensors is reduced during the whole process of the game.

Figure 32 shows the performance of the game when two rules *all C* and *all D* are used together with an other rule as follows: kD (see Figure 32a,b), kC (see Figure 32c,d), or kDC (see Figure 32e,f). We can also compare these results with ones obtained for WSN 45 (see Figure 21). The behavior of the considered set of rules for both instances is very similar—for all subsets of rules, the system achieves high-quality solutions. We can see only some difference in the behavior of the subset *all C*, *all D*, and kD as follows: in the case of WSN 125, the system needs some time to obtain a suboptimal number of sensors turned on.

### 9.6. LA-Based Approach: Instances WSN 100 Rand, WSN 200 Rand, and WSN 500 Rand

The purpose of this set of experiments was to observe behavior of the algorithm for instances with randomly selected locations of sensors. This will be achieved by using WSN 100 rand, WSN 200 rand, and WSN 500 rand, and some comparisons will be performed with instance WSN 125, based on the deterministic locations of the sensors.

**Figure 33:**
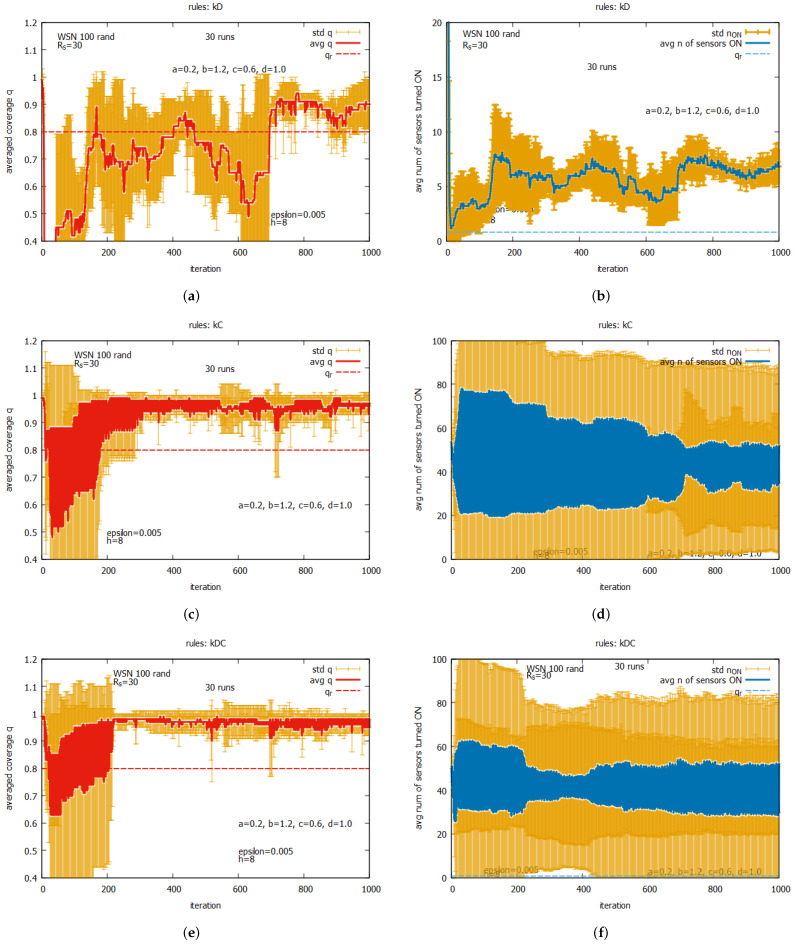
WSN 100 rand: averaged value of (**a**) coverage *q* for rule kD, (**b**) the number n_on of sensors turned on for rule kD, (**c**) coverage *q* for rule kC, (**d**) the number n_on of sensors turned on for rule kC, (**e**) coverage *q* for rule kDC, (**f**) the number n_on of sensors turned on for rule kDC.

**Figure 34:**
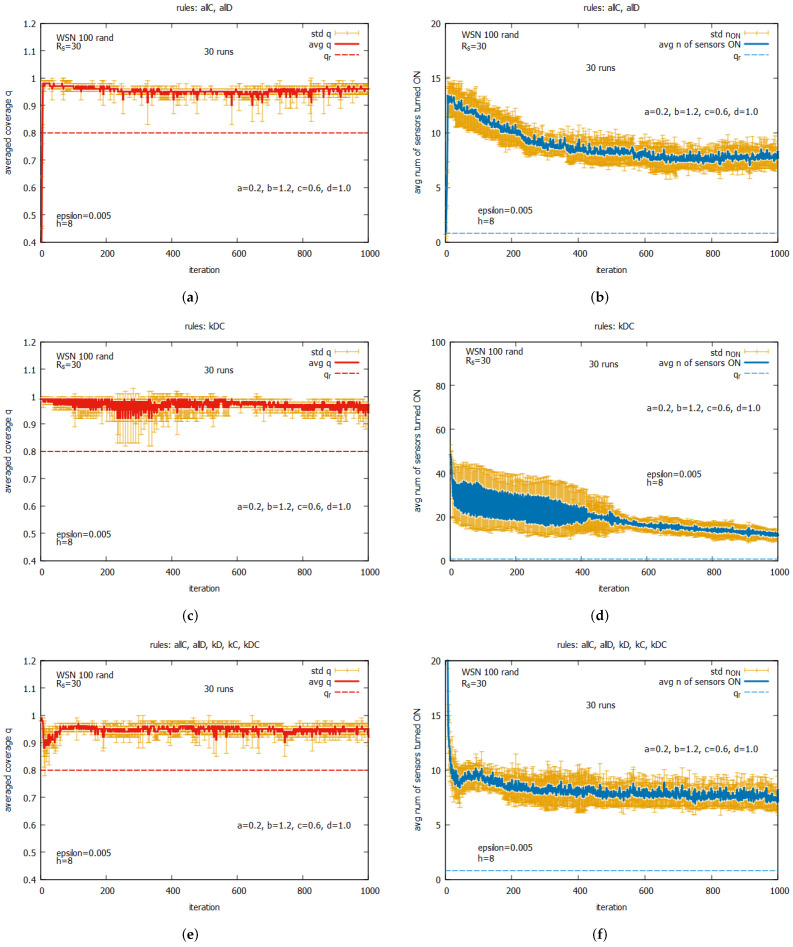
WSN 100 rand: averaged value of (**a**) coverage *q* for rules {all C,all D}; (**b**) the number n_on of sensors turned on for rules {all C,all D}; (**c**) coverage *q* for rules {kD,kC,kDC}; (**d**) the number n_on of sensors turned on for rules {kD,kC,kDC}; (**e**) coverage *q* for rules {all C,all D,kD,kC,kDC}; (**f**) the number n_on of sensors turned on for rules {all C,all D,kD,kC,kDC}; (**d**) the number n_on.

**Figure 35:**
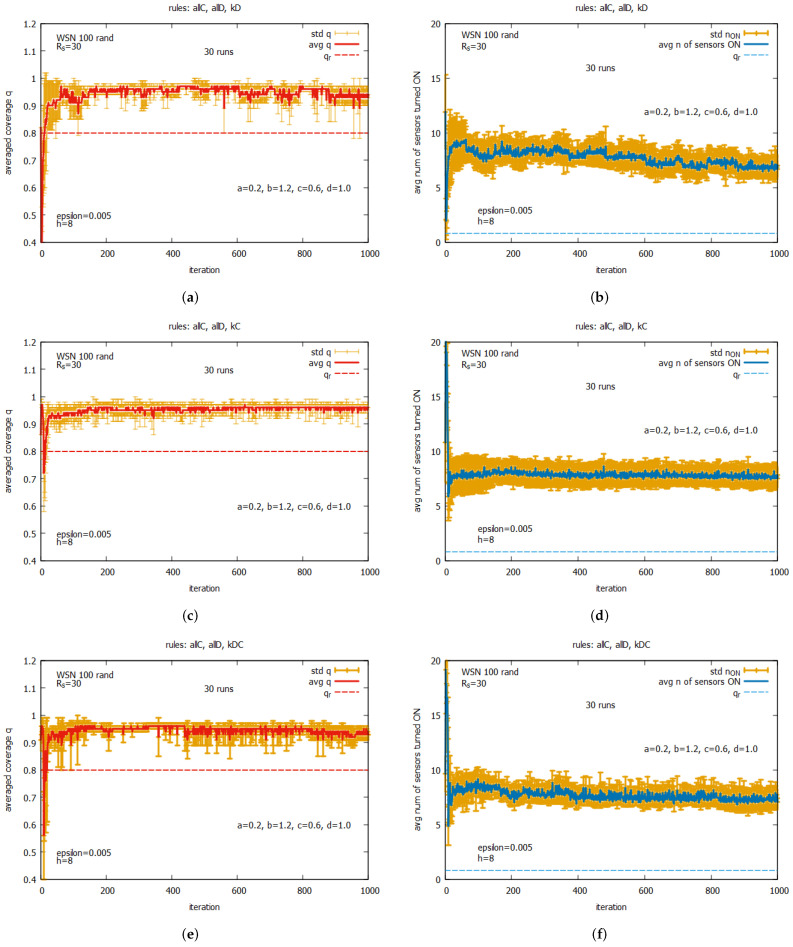
WSN 100 rand: averaged value of (**a**) coverage *q* for rules {all C,all D,kD}; (**b**) the number n_on of sensors turned on for rules {all C,all D,kD}; (**c**) coverage *q* for rules {all C,all D,kC}; (**d**) the number n_on of sensors turned on for rules {all C,all D,kC}; (**e**) coverage *q* for rules {all C,all D,kDC}; (**f**) the number n_on of sensors turned on for rules {all C,all D,kDC}.

The averaged results of experiments for WSN 100 rand are presented at Figure 33, Figure 34 and Figure 35. Figure 33 presents results assuming that only one from rules kD, kC, and kDC is used, and we may compare these results with the ones obtained for WSN 125 (see Figure 30). We can observe that despite the different ways followed to create both instances, the results are very similar.

Figure 34 presents the results for the set of rules *all C* and *all D* (see Figure 34a,b); for the set kD, kC, and kDC, see Figure 34c,d); and for the whole set of five rules (see Figure 34e,f). Again, we can compare these results with the ones obtained for WSN 125 (see Figure 31). One can see that the results for both instances are again similar, except for the case using subset kD, kC, and kDC, where we see some temporary increase in the average value and standard deviation of the averaged values of n_on.

Figure 35 shows the performance of the game when two rules, *all C* and *all D*, are used together with an other rule as follows: kD (see Figure 35a,b), kC (see Figure 35c,d) or kDC (see Figure 35e,f). We can also compare these results with the ones obtained for WSN 125 (see Figure 32). The behavior of the considered set of rules for both instances is near identical. Reassuming this part of the experiments, we can judge that a deterministic or random location of the approximate number of sensors in a monitored area does not influence the performance of the self-organized algorithm aiming to solve the coverage problem.

**Figure 36:**
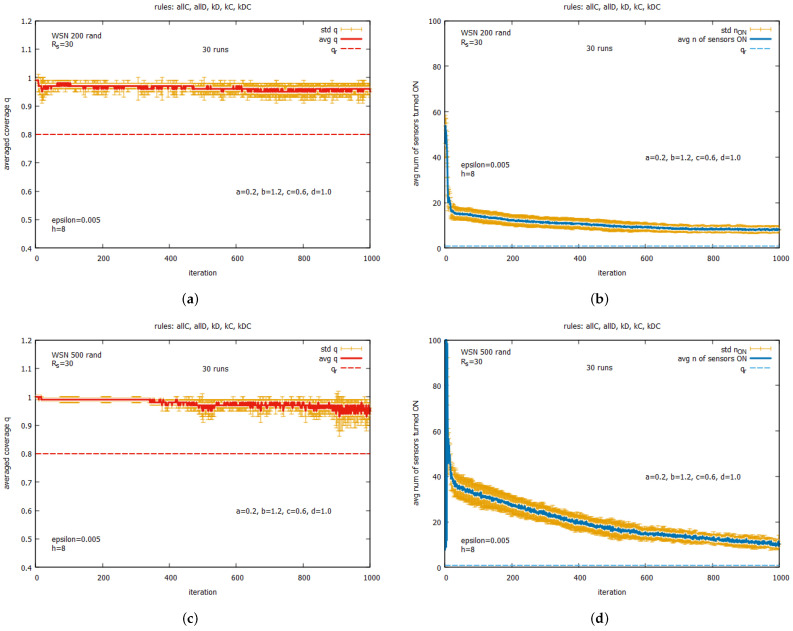
WSN 200 rand and WSN 500 rand with the set of rules {all C,all D,kD,kC,kDC}: averaged value of (**a**) coverage *q* for WSN 200 rand, (**b**) the number n_on of sensors turned on for WSN 200 rand, averaged value of (**c**) coverage *q* for WSN 500 rand, (**d**) the number n_on of sensors turned on for WSN 500 rand.

Figure 36 presents the results of the experiments for WSN 200 rand and WSN 500 rand. In both these instances, the same set of rules {all C,all D,kD,kC,kDC} was used. One can see that for both these instances, the values of *q* very quickly reach very high values close to 1. In the beginning, the number of sensors turned on is relatively high, but this decreases in the subsequent iterations of the algorithm. The main difference between runs of the algorithm for both instances is the speed of reducing the number of sensors turned on. We can see that, with an increase in the number of sensors at one instance, the speed of reducing the number of sensors turned on decreases.

### 9.7. Discussion of Experimental Results

In this Section, we present the experimental results demonstrating the performance of the self-organizing LA-based and CA-based algorithms for solving the coverage problem in WSN. The experiments were conducted for a wide range of WSN instances and different subsets of rules. TWe primarily focused on the LA-based algorithm, which is the primary novelty of this paper.

The purpose of the experiment using the WSN 5 instance (see Section 9.2) was to provide insights into the operation of the LA-based algorithm, illustrating the effect of individual steps of the algorithm on individual LA-based agents. Special attention was paid to the issue of rule selection by agents, which occurs with a high probability 1−ϵ by retrieving the best performing rule from the memory over the last *h* time steps, or sometimes, with a probability ϵ, by randomly selecting a rule from the set of available rules.

It is worth noting that the studied self-organizing multi-agent-based algorithms are parallel and probabilistic. Their convergence to solutions is a complex random process that cannot be easily observed or interpreted as a sequence of parallel actions performed by agents. Instead, it results from the coincidence of collective actions, with the probability of success increasing if certain conditions related to algorithm parameters are met. In the experiment with WSN 5, we observed an example of this type of coincidence created by the agents’ rules. Observing such coincidences is possible only for very small instances of WSNs. For such small instances, formal analysis methods, such as Markov Chain models, can potentially be applied. For larger instances, we can rely only on simulation methods, and our further discussion is based on statistical data obtained from experiments.

The search engine of both studied versions of the algorithms consists of the following two main components: a subset of rules available to agents in a given run and the mechanism for selecting a rule by an agent in the current time step. The selected rules change the battery states of the controlled sensors, and these states are used by agent-players as actions in a game with neighboring players. The rules can be considered building blocks that agents use in a competitive game to construct a part of a global solution. Each of the considered rules possesses different abilities to contribute to the process of building a global solution. These abilities can also depend on the specific problem being solved. The second component of the algorithms, responsible for rule selection, manages the process used to maximize the reward (payoff) obtained in the game. The selection process is based on learning, which involves storing the results of the game obtained in subsequent iterations. We considered two options for learning as follows: vertical learning (LA-based systems) and horizontal learning (CA-based systems).

The results of the experiments using the LA-based system have been shown for a wide range of the number of sensors in instances where, while single rules kD, kC, and kDC, provide coverage levels exceeding $q_{r}$, only rule kD maintains a reasonable number of sensors turned on, averaging at around six. For WSN 45, it requires an average of around 600 iterations. However, for WSNs with a higher number of sensors, the values of *q* can fluctuate around $q_{r}$. The best performance is observed for the pair of rules all C and all D. This algorithm requires an average of around 50–100 iterations to reach a global solution, with an average value of n_on being around 7, and with low standard deviation values. Similar results can also be obtained when this pair of rules works collectively with the rules kD, kC, and kDC, or subsets of these rules.

The results of the experiments using instances with higher numbers of sensors (up to 500 sensors) generally confirm the observations obtained from previous experiments. We can see that the algorithm achieves, in the first iterations, the level of coverage *q* meeting the requirements, but it is accompanied by a relatively high number of sensors turned on. With an increase in the iterations of the algorithm, we can see a decrease in the number of sensors turned on. We can observe two phases of this decrease. In the first phase, which lasts around 20–30 iterations, the number of sensors turned on is reduced very quickly. In the second phase, the speed of reducing the number of sensors turned on significantly depends on the size of the instance.

The results of the experiments conducted using the CA-based system with WSN 45 have shown that only the pair all C and all D provides results close to optimal, which depend on the values of the parameter pstratmut. The optimal results are obtained much faster than in the case of the LA-based system, but the main disadvantage of the CA-based system is the relatively large values of standard deviations. Results presented in [9] show that the values of standard deviations decrease with an increase in the number of sensors in an instance.

While the results presented in this paper show that the best building block for solving the coverage problem in WSN corresponds to the pair of rules all C, all D, our recent study [50] suggests that the choice of the best building block and the choice of vertical or horizontal learning for selecting such building blocks depend on the problem to be solved. In the mentioned study, we applied the same adaptive CA-based approach and the same set of rules to solve the pattern formation problem in 2D space. The obtained results show that the CA-based approach works very well for this problem, and the best building block is represented either by the kC or kD rule, or by a percentage combination of these two rules.

## 10. Conclusions

In this paper, we considered the issue of developing self-organizing algorithms aimed at solving the coverage problem in WSNs. For this purpose, we used a game-theoretical framework based on the application of a variant of the spatial Prisoner’s Dilemma game. This framework was used to build a multi-agent system, where agent-players in the process of iterated games wish to achieve a Nash equilibrium point, providing them the maximal values of payoffs. An equilibrium reached corresponds to a global solution for the coverage problem represented by two objectives as follows: a coverage and the corresponding number of sensors that are turned on. A multi-agent system using the game-theoretic framework assumes the creation of a graph model of the WSN and the further interpretation of the WSN graph as agents participating in iterated games.

We considered two models of agents as follows: we proposed, in this paper, a model based on the application of (ϵ,h)-learning automata, and have earlier proposed a model based on the application of adaptive cellular automata. In both cases, we dealt with processes of learning and adaptation by agents in a WSN environment. The ways of learning and adaptation are different and, therefore, we call them verticallearning (LA-based agents) or horizontallearning (CA-based agents).

We performed a number of experiments, using different WSN instances and two variants of self-organizing systems, demonstrating their performance for different system parameters. We have shown that, while both self-organizing algorithms are able to reach in a fully distributed way—without any global coordinator—a global solution, the better performance is provided by LA-based systems. Such systems provide higher values of the coverage, using a lower number of sensors that need to be turned on, and the solutions are characterized by much lower standard deviation values.

This paper presents self-organized algorithms for solving the coverage problem, opening new avenues for maximizing the lifetime of WSN by repeatedly executing the procedure of self-organized coverage until the WSN batteries reach a state where further charging is not possible. Our future work will focus on developing a new class of self-organizing algorithms that solves the problem of maximizing the lifetime of WSNs on the fly.

## Figures and Tables

**Table 1:** Payoff function of SPD-like game for coverage optimization problem.

*i*-th Agent’s Action	Fulfilment of $q_{r}^{i}$	
Turn on battery (C)	$q_{curr}^{i-off}\geq q_{r}^{i}$	
	no	yes
	$rew_{i}^{on+}=d$	$rew_{i}^{on-}=c$
Turn off battery (D)	$q_{curr}^{i}\geq q_{r}^{i}$	
	no	yes
	$rew_{i}^{off-}=a$	$rew_{i}^{off+}=b$

## Data Availability

Data are contained within the article.
